# Novel pyrazoline and pyrazole “turn on” fluorescent sensors selective for Zn^2+^/Cd^2+^ at *λ*_em_ 480 nm and Fe^3+^/Fe^2+^ at *λ*_em_ 465 nm in MeCN†

**DOI:** 10.1039/d4ra00036f

**Published:** 2024-01-22

**Authors:** Alexander Ciupa

**Affiliations:** a Materials Innovation Factory, University of Liverpool 51 Oxford Street Liverpool L7 3NY UK ciupa@liverpool.ac.uk

## Abstract

A small series of simple pyrazoline and pyrazole based sensors, all derived from the same chalcone precursors, were synthesised, characterised and screened for their fluorescence “turn on” properties in the presence of multiple metals. Pyrazole 8 displayed an excellent fluorescence profile with approx. 20× fold increase in *λ*_em_ 480 nm with Zn^2+^ compared to a 2.5× fold increase with Cd^2+^. Pyrazole 9 displayed a 30× fold increase at *λ*_em_ 465 nm for Fe^3+^ compared to Fe^2+^ with a Fe^3+^ limit of detection of 0.025 μM. The corresponding pyrazolines displayed contrasting properties with important implications for future pyrazoline and pyrazole sensor design.

## Abstract 2

A small series of simple pyrazoline and pyrazole based sensors, all derived from the same chalcone precursors, were synthesised, characterised and screened for their fluorescence “turn on” properties in the presence of multiple metals.

## Graphical abstract

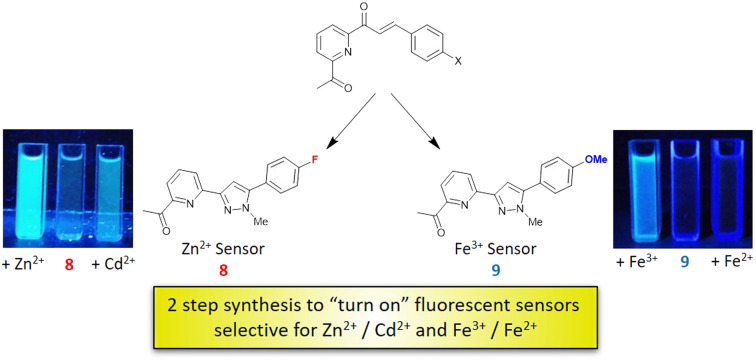

## 1 Introduction

**Fig. 1:**
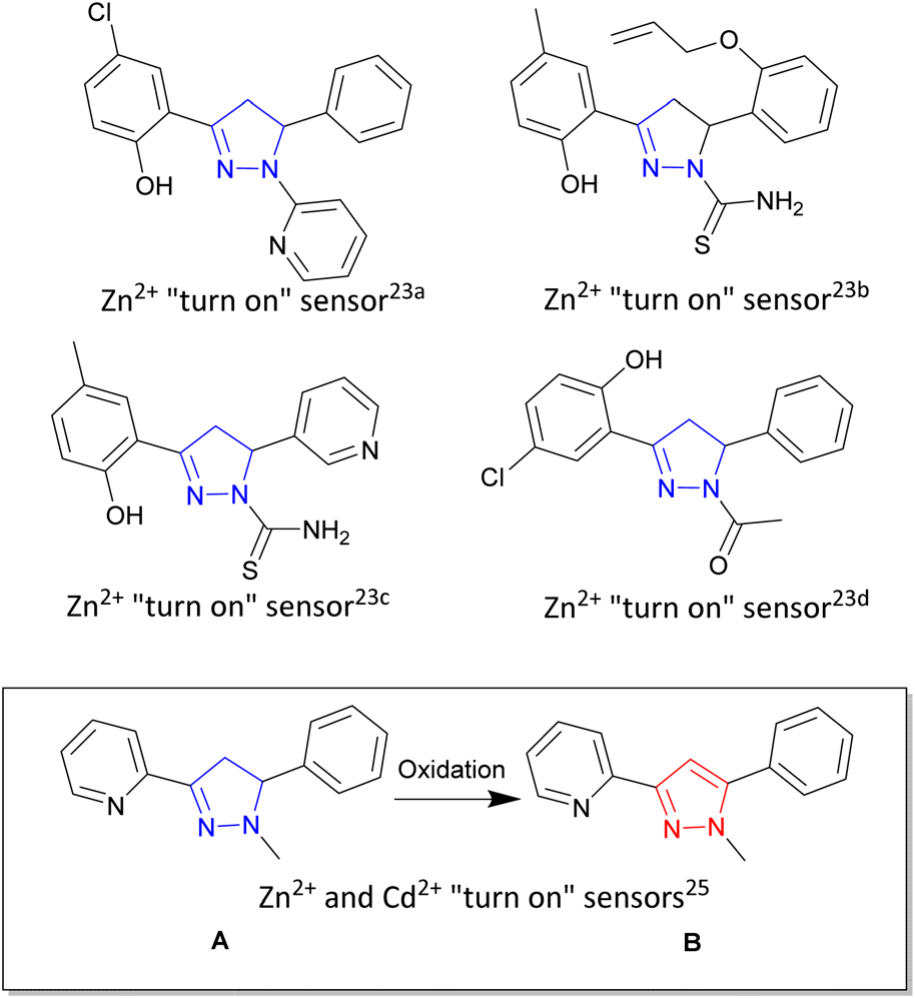
Pyrazoline heterocycle (shown in blue) in fluorescent “turn on” and “turn off” sensors developed by Miao and Zhao groups.^23^ Inset demonstrates “turn on” fluorescent sensor pyrazoline A can be converted into pyrazole B with different photophysical properties.^25^

Zinc is the second most abundant transition metal in the human body^1^ critical to a diverse range of biological functions including enzyme maintenance,^2^ gene expression^3^ and neurological functions.^4^ Unregulated zinc is implicated in a number of biological illnesses ranging from Alzheimer's disease,^5^ epilepsy^6^ and Parkinson's disease.^7^ Cadmium, also a group 12 transition metal sitting beneath zinc in the periodic table, is a highly toxic environmental and industrial pollutant with long term exposure linked to renal, breast and lung cancers.^8,9^ An ideal analytical technique to track and trace these two metals in biological systems is fluorescence spectroscopy due to its high specificity, low limit of detection and ability to fine-tune the emission wavelength.^10–12^ Fluorescence sensing typically involves two common approaches, either a “turn on” sensor^13,14^ in which the presence of the analyte of interest increases fluorescence emission intensity (*λ*_em_) or a “turn off” sensor^15,16^ in which the analyte decreases fluorescence intensity. A major challenge remains which is the ability to selectively detect zinc over cadmium and *vice versa* in complex mixtures, with only a few examples having been reported in the literature.^17^ Multiple heterocyclic scaffolds are used to tune metal ion selection, photophysical and chemical properties to meet a particular sensor requirement. Pyrazoline,^18^ a 5 membered heterocyclic ring, and the closely related pyrazole^19^ are leading examples combining the advantageous properties of modular design from commercially available starting materials and the ability to Taylor the orientation of the three branching units off the main pyrazoline or pyrazole core (shown in blue and red respectively in Fig. 1). Pyrazolines chelators for gold,^20^ tin^21^ and ruthenium^22^ have all been reported with the groups of Miao and Zhao pioneering both “turn on” and “turn off” pyrazoline fluorescent sensors for multiple systems including Zn^2+^ in aqueous environments and living systems.^23^ A variety of photophysical processes result in increased fluorescence emission^18,19^ with the blocking of the photoinduced electron transfer (PET) process upon Zn^2+^ chelation a common pathway.^13,17*a*,23*a*,35^ Mixed fluorescent sensors composed of both pyrazoline and pyrazole units have also been reported^24^ highlighting that structural complexity is not a prerequisite for complex functionality, simple molecular structures provide the tools to detect biologically important analytes in living systems.

Previous work^25^ reported simple pyridine pyrazoline A as a “turn on” fluorescent sensor for both Zn^2+^ and Cd^2+^ producing a 1.7× fold higher response for Cd^2+^/Zn^2+^ (Fig. 1 inset). Oxidation of A to the corresponding pyrazole B produced a “turn on” sensor capable of distinguishing Zn^2+^/Cd^2+^ with *λ*_em_ 380 nm and *λ*_em_ 350 nm respectively. Herein we report the next generation of sensors with three novel pyrazolines 4–6 and their related three novel pyrazoles 7–9 with pyrazole 8 able to detect Zn^2+^ with a 20× fold increase in fluorescence at *λ*_em_ 480 nm compared to absence of Zn^2+^. Cd^2+^ resulted in only a 2.5× fold increase in fluorescence at *λ*_em_ 480 nm. In contrast, pyrazole 9 displayed only a minor increase in fluorescence with Zn^2+^ and Cd^2+^ but 30× fold increase in *λ*_em_ 465 nm with Fe^3+^ but surprisingly not Fe^2+^. These results within provide valuable insight for the design of the next generation of pyrazoline and pyrazole fluorescent sensors specific for Zn^2+^ and Fe^3+^.

## 2 Results and discussion

**Scheme 1:**
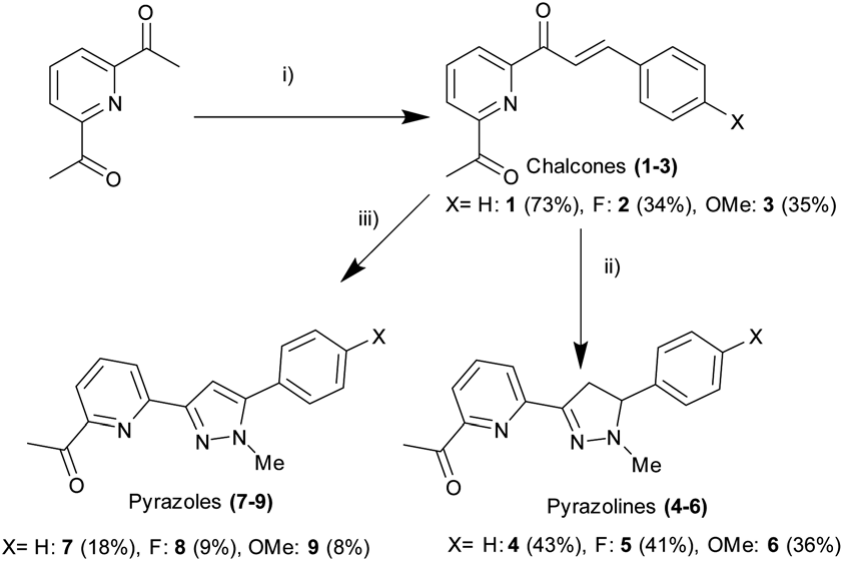
Synthesis of pyrazolines 4–6 and pyrazoles 7–9 from chalcone precursors 1–3. (i) 0.5 eq. of substituted aldehyde, 0.5 eq. NaOH, MeOH, rt, 18 h (ii) 1.2 eq. H_2_NNHMe, MeOH, rt, 3 h (iii) 8 eq. H_2_NNHMe, MeOH, rt, 24 h. Chemical yields are displayed in brackets.

2,6-Diacetylpyridine underwent Claisen–Schmidt condensation^26^ with the required substituted aromatic aldehyde to afford the chalcone precursors 1–3 in acceptable yield (34–73%). After extensive investigation, it was discovered that a 2 : 1 ratio of ketone to aldehyde with catalytic amount of NaOH promoted formation of the required mono-chalcone and minimal formation of the bis-chalcone side product (ESI S3†). Bis-chalcone could be easily removed by gravity filtration allowing the desired chalcone to be further purified by recrystallisation preventing the requirement for time consuming column chromatography. Conversion of chalcone into pyrazoline 4–6 was achieved (ESI S2†) by adapting previous methods^25,27^ involving the 1,2 addition of methylhydrazine in slight excessive at room temperature (36–43% yield). Hydrazone formation was possible however following an extensive aqueous wash only the desired product was observed, this is comparable with other reports of hydrazone hydrolysis under similar conditions in the literature (Scheme 1).^31^

This method was modified to yield the closely related pyrazole series 7–9 using excess methylhydrazine and longer reaction time to afford the pyrazole series in satisfactory yield (8–18%). Oxidation of pyrazoline to pyrazole has been reported previously^25,28^ with only a few literature examples of direct transformation from chalcone to pyrazole^29,30^ typically involving a catalyst and/or heating. One pot pyrazole synthesis from chalcone using excess methylhydrazine was confirmed by ^1^H NMR spectroscopy with formation of an aromatic pyrazole ^1^H singlet signal at approx. 7.1 ppm (H^d^ in Fig. 4 for 7, ESI S3† for 6 and 9) with the absence of the three sets of doublet of doublet signals characteristic of a pyrazoline ring reported previously.^25,27^ High resolution mass spectrometry confirmed pyrazole formation. The photophysical properties of these six novel compounds was explored using well-established protocols in the literature.^12–20,23,25^

**Fig. 2:**
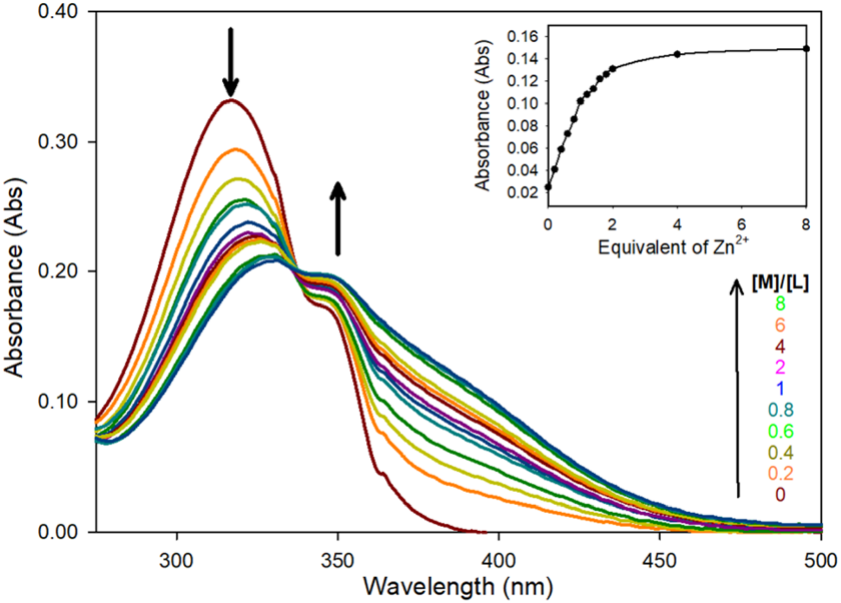
Absorbance spectra of 5 (20 μM) with incremental Zn^2+^ in MeCN.

Zn^2+^ chelation for the pyrazoline series was confirmed using UV/Vis spectroscopy in MeCN, this solvent was selected to enable direct comparison with previous studies^25^ and to determine the optimum photophysical properties for a future water-soluble sensor. The initial absorbance band at 320 nm (*ε* = 16 550 M^−1^ cm^−1^) decreasing with the linear appearance of a new band at 340 nm up to 2.0 equivalents (eq.) Zn^2+^ (Fig. 2 for pyrazoline 5 and ESI S4† for 4 and 6). All three pyrazolines exhibited similar absorbance profiles suggesting substitution at the aryl ring was not detrimental to chelation.

**Fig. 3:**
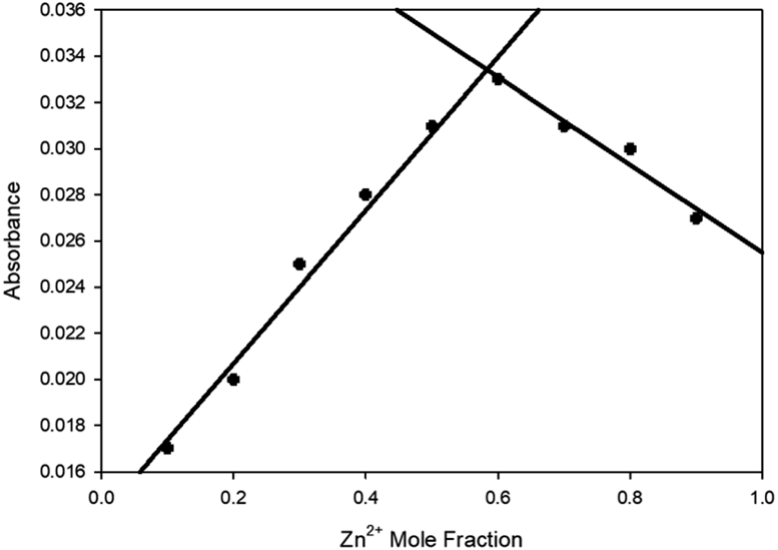
Job plot^32^ analysis of 5 with varying Zn^2+^ mole fractions in MeCN with absorbance at 400 nm, [Zn^2+^] + [5] = 100 μM.

A 1 : 2 ratio of sensor to Zn^2+^ was suggested from the linear increase in absorbance at 375 nm observed up to 2 eq. of Zn^2+^ (Fig. 2 inset) with further increases producing no significant increase in absorbance. Job plot analysis^32^ (Fig. 3 for 5 ESI S5† for 4–9) also indicated a 1 : 2 ratio between all pyrazolines and Zn^2+^. This is in contrast to pyrazoline A which had a 1 : 1 ratio of sensor to Zn^2+^. The addition of the acetyl group may be responsible for this additional Zn^2+^ chelation.

**Fig. 4:**
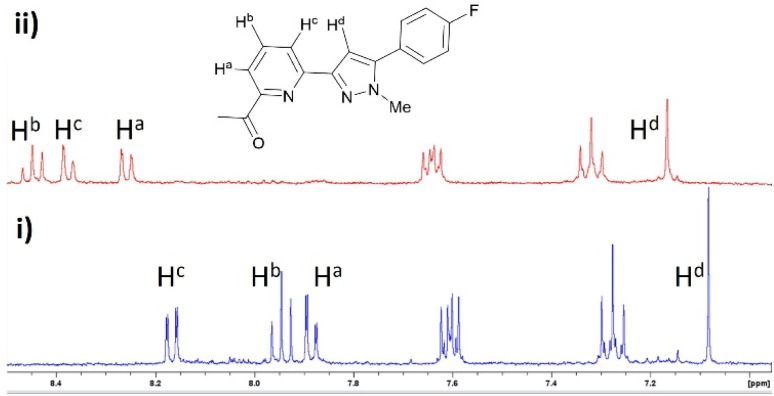
Partial ^1^H NMR spectra of (i) pyrazole 8 (MeCN-d_3_, 2 μM) and (ii) with addition of 2 eq. Zn^2+^.

Pyrazole 8 was selected for a Zn^2+ 1^H NMR spectroscopy titration experiment to confirm Zn^2+^ chelation (Fig. 4). Addition of 2 eq. Zn^2+^ resulted in significant shifts for H^a^ and H^c^ doublet of doublet peaks on the pyridine ring at 7.88 ppm and 8.17 ppm downfield to 8.26 ppm and 8.37 ppm. The H^b^ triplet pyridine proton peak and H^d^ singlet pyrazole peak increased to higher chemical shift from 7.95 ppm and 7.08 ppm to 8.45 ppm and 7.17 ppm consistent with Zn^2+^ chelation and previous literature examples.^14,17,25,31^ A similar response was observed with pyrazole 7 and pyrazoline compounds 4–6 (see ESI S2†) suggesting the pyridine ring chelates Zn^2+^ regardless if connected to a pyrazoline or pyrazole heterocycle.

**Fig. 5:**
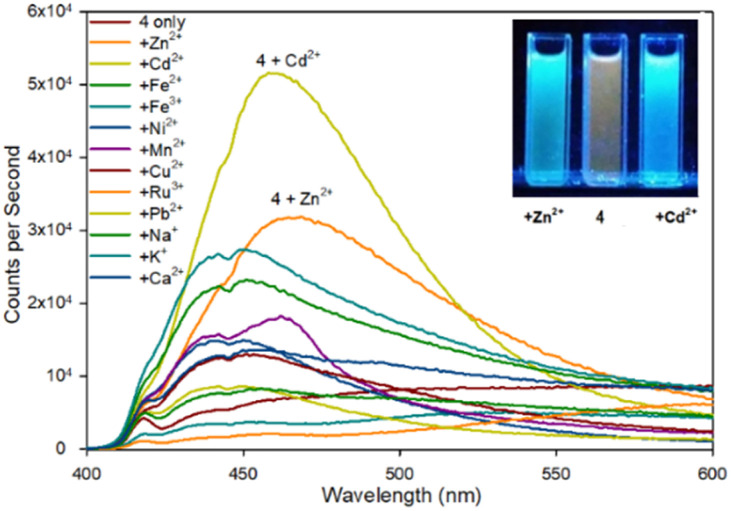
Fluorescence spectra of pyrazoline 4 upon addition of 5 eq. of the indicated metal. Inset from left to right, +Zn^2+^, 4 only, +Cd^2+^, *λ*_ex_365 nm 10 W lamp, 100 μM 4 with 500 μM metal cation.

Fluorescence spectroscopy was used to screen multiple metals to determine useful fluorescence properties. Pyrazoline 4 remains more sensitive to Cd^2+^ than Zn^2+^ with a 7.3× fold increase at *λ*_em_ 480 nm compared to 4.5× fold with Zn^2+^ (Fig. 5). It is interesting to note the similarity with the previous reported sensor A^25^ lacking the acetyl group also displayed higher fluorescence with Cd^2+^ over Zn^2+^ with a ratio of 1.7× fold higher emission at the same *λ*_em_ 465 nm with Cd^2+^ over Zn^2+^, comparable to the 1.6× fold reported above. A Zn^2+^ limit of detection (LoD)^33^ of 0.0319 μM for 4 and 0.010 μM for 5 was calculated (see ESI S7†) which is similar to the 0.0202 μM Zn^2+^ LoD for A^25^ other Zn^2+^ based “turn on” fluorescent sensors.^23*b*^ This suggests the additional acetyl group is not conveying any beneficial effect in terms of Zn^2+^/Cd^2+^ analyte selectivity but provides a slight improvement in fluorescence response. This should be factored into future pyrazoline based sensor design. It is noteworthy to highlight further chemical modification to introduce additional functional groups *via* the acetyl group may enhance “turn on” fluorescence properties and this is ongoing research in our laboratory.

**Fig. 6:**
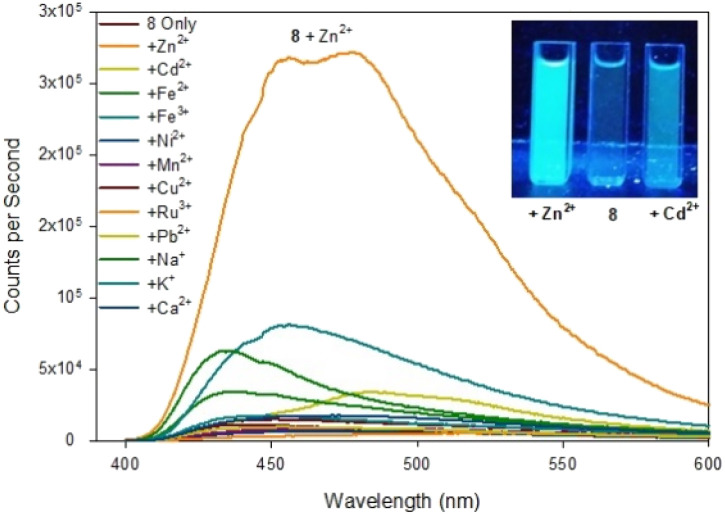
Fluorescence spectra of pyrazole 8 upon addition of 5 eq. of the indicated metal. Inset from left to right, +Zn^2+^, 8 only, +Cd^2+^, *λ*_ex_365 nm 100 W lamp, 100 μM 8 with 500 μM metal cation.

Previous studies demonstrated pyrazolines can be converted into the closely related pyrazoles displaying “turn on” fluorescence properties in the presence of different cations. To investigate further, pyrazole series 7–9 were screened across multiple metals and to our surprise pyrazole 8 demonstrated an excellent “turn on” fluorescence response for Zn^2+^/Cd^2+^ (Fig. 6 for 8, ESI S6† for 7).

**Fig. 7:**
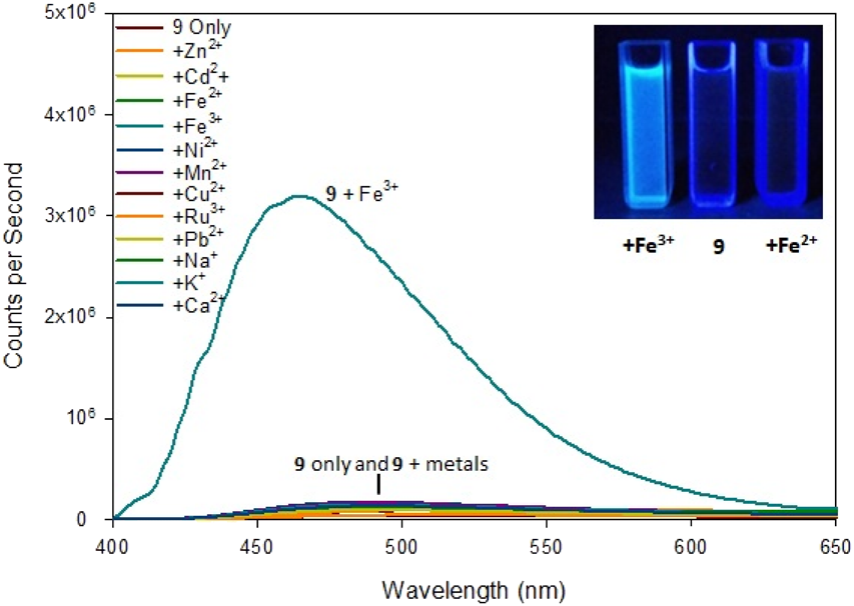
Fluorescence spectra of pyrazoline 9 on addition of 5 eq. of the indicated metal. Inset from left to right, +Fe^3+^, 9 only, +Fe^2+^ with *λ*_ex_ 254 nm 6 W lamp. 100 μM 9 with 500 μM metal cations.

Upon addition of 5 eq. Zn^2+^ the fluorescence at *λ*_em_ 480 nm increased 20× fold whereas the addition of 5 eq. Cd^2+^ only resulted in a 2.5× fold increase at *λ*_em_ 480 mn resulting in approximately 8× fold increase in selectivity for Zn^2+^ over Cd^2+^. This is in contrast to the previously reported pyrazole B^25^ lacking the acetyl group which displayed a 13× fold increase *λ*_em_ 380 nm upon addition of 5 eq. Zn^2+^. Job plot analysis (ESI S5†) suggests a 1 : 1 ratio between 8 and Zn^2+^ similar to pyrazole B reported previously.^25^ Pyrazole 7 did not display a significant increase in fluorescence on addition of Zn^2+^ or Cd^2+^ (see ESI S6†) suggesting the electronegative fluorine group on the aryl ring was a key requirement of this “turn on” fluorescent response. Substitution of the 4-F for an electron donating 4-OMe group resulted in 9 which abolished this Zn^2+^ response. Further investigation revealed 9 displayed a 30× fold increase at *λ*_em_ 465 mn with Fe^3+^ but not Fe^2+^ (Fig. 7).

**Fig. 8:**
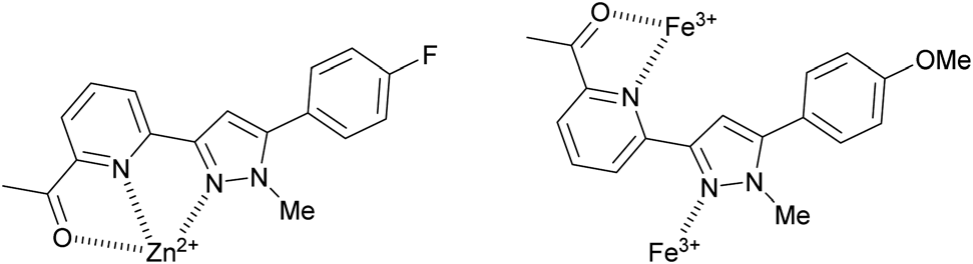
Proposed binding mechanisms for pyrazole 8 with Zn^2+^ and 9 with Fe^3+^.

The difference was profound and could be visibly observed using a low power 6 W *λ*_ex_ 254 nm TLC lamp (Fig. 7 inset). Job plot analysis (ESI S5†) suggested a 1 : 2 sensor to Fe^3+^ ratio. Pyrazole 9 is an excellent candidate for a “turn on” fluorescent sensor with a calculated Fe^3+^ LoD of 0.025 μM. This is noteworthy as the first reported Fe^3+^ specific pyrazoline sensor had a Fe^3+^ LoD of 3 μM (ref. 34) with recent pyrazole based Fe^3+^ sensors reporting Fe^3+^ LoD ranging from 0.021 μM (ref. 35) to 0.0004 μM.^24^ The proposes 1 : 1 binding mechanism of pyrazole 8 with Zn^2+^ and a 1 : 2 binding mechanism of pyrazole 9 with Fe^3+^ is displayed in Fig. 8. This agrees with previously reported pyrazoles with Zn^2+^ and Fe^3+^ in the literature.^19,36–38^

**Fig. 9:**
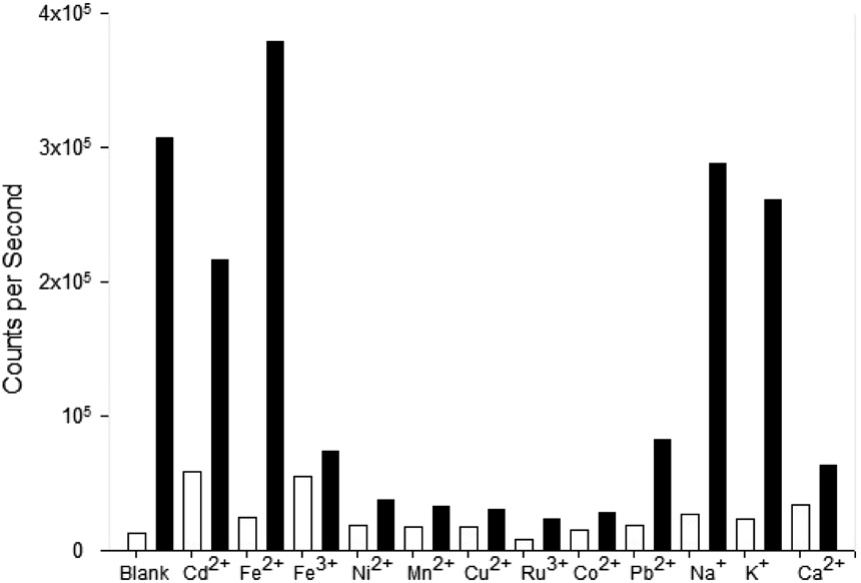
Competition experiments for pyrazole 8. The white bar represents 8 (MeCN, 20 μM, *λ*_ex_ 290 nm, *λ*_em_ = 480 nm) with 5 eq. of the indicated cation; the black bars is the same plus 5 eq. Zn^2+^ after equilibrating for 3 min.

Competition assays were performed to assess pyrazole 8 Zn^2+^ “turn on” fluorescence response in the presence of competing metal cations (Fig. 9). Fluorescence quenching was observed upon addition of a range of paramagnetic metals including Fe^3+^, Ni^2+^ and Co^2+^, a common phenomenon observed in the literature.^25^ A good fluorescence response in the presence of Na^+^ and K^+^ cations was observed suggesting the presence of these singularly charged metals was not detrimental to the Zn^2+^ sensing properties of 8.

**Fig. 10:**
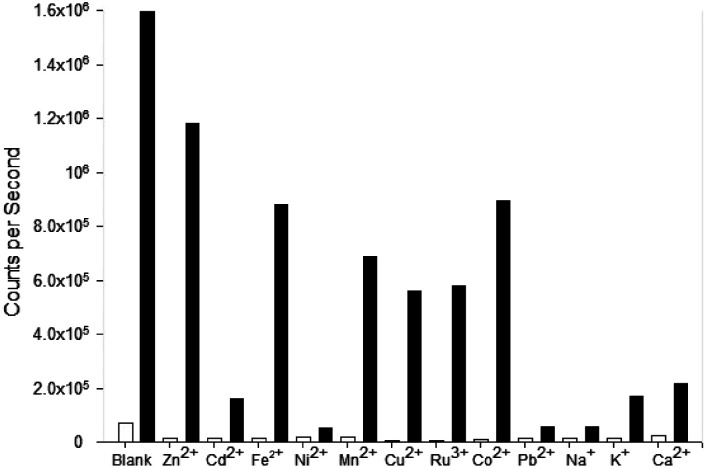
Competition experiments for pyrazole 9. The white bar represents 9 (MeCN, 20 μM, *λ*_ex_ 290 nm, *λ*_em_ = 455 nm) with 5 eq. of the indicated cation; the black bars is the same plus 5 eq. Fe^3+^ after equilibrating for 3 min.

Pyrazole 9 was also submitted to a competition assay to evaluate its ability as a Fe^3+^ “turn on” sensor and it displayed a slightly better profile than 8 retaining modest fluorescence response in the presence of Fe^2+^, Mn^2+^, Cu^2+^, Ru^3+^ and Co^2+^ (Fig. 10). Unfortunately, the presence of Na^+^ and K^+^ did significantly reduce *λ*_em_ 455 nm fluorescence in contrast to 8.

The competition assay profiles for 8 and 9 are similar to previously reported pyrazolines with paramagnetic metals typically hampering fluorescent response.^25^ Further work is required to enhance analyte chelation and prevent competing cations from disrupting the “turn on” response. The unexpected switch from a Zn^2+^ to a Fe^3+^ “turn on” sensor for 8 and 9 highlight the modular nature of the pyrazole heterocycle and how small modifications can have a profound influence on photophysical properties.

## 3 Conclusions

The addition of an acetyl group on the pyridine of pyrazole sensor 8 improved fluorescence properties (20× fold increase at *λ*_em_ 480 nm) for the detection of Zn^2+^ over Cd^2+^(2.5× fold increase also at *λ*_em_ 480 nm) compared to sensor B^25^ reported previously. Substitution of the electronegative 4-F on pyrazole 8 for an electron donating 4-OMe group in 9 resulted in the unexpected discovery of a “turn on” fluorescent sensor for Fe^3+^ at *λ*_em_ 465 nm. 9 displayed a Fe^3+^ LoD of 0.025 μM which is comparable to recently reported Fe^3+^ fluorescent sensors.^24,35^ These results suggest the acetyl group is highly beneficial and should be factored into future pyrazole sensor design. In contrast the same modification to pyrazoline sensors conferred no significant advantage in selectivity towards Zn^2+^/Cd^2+^ compared to sensor A^25^ however it did produce a slight increase in Zn^2+^ LoD. The above studies were performed in MeCN to aid comparison to previous work which was also carried out in MeCN. Previous exploratory reports for Zn^2+^ fluorescent sensors were performed in pure organic solvents also including MeOH,^39,40^ THF,^41,42^ DMF^43^ and DMSO.^44,45^ The results within provide a firm foundation for developing aqueous based sensors for lead pyrazole 8 with Zn^2+^ and 9 with Fe^3+^, this is ongoing work and will be reported in due course.

## Author contributions

Alexander Ciupa designed, synthesised and characterised all compounds, performed all UV/Vis, NMR and fluorescence spectroscopy experiments and authored the manuscript.

## Conflicts of interest

There are no conflicts to declare.

## Supplementary Material

RA-014-D4RA00036F-s001

## References

[cit1] Chasapis C. T., Spiliopoulou C. A., Loutsidou A. C., Stefanidou M. E. (2012). Arch. Toxicol..

[cit2] Andreini C., Bertini I. (2012). J. Inorg. Biochem..

[cit3] O'Halloran T. V. (1993). Science.

[cit4] Frederickson C. J. (2001). BioMetals.

[cit5] Lovell M. A., Robertson J. D., Teesdale W. J., Campbell J. L., Markesbery W. R. J. (1998). J. Neurol. Sci..

[cit6] Doboszewska U., Młyniec K., Wlaz A., Poleszak E., Nowak G., Wlaz P. (2019). Pharmacol. Ther..

[cit7] Sikora J., Ouagazzal A. M. (2021). Int. J. Mol. Sci..

[cit8] Järup L. (2003). Br. Med. Bull..

[cit9] Genchi G., Sinicropi S. M., Lauria G., Carocci A., Catalano A. (2020). Int. J. Environ. Res. Public Health.

[cit10] Kim H. N., Ren W. X., Kim J. S., Yoon J. (2012). Chem. Soc. Rev..

[cit11] Chen Y., Bai Y., Han Z., He W., Guo Z. (2015). Chem. Soc. Rev..

[cit12] Xu L., Xu Y., Yhu W., Yang C., Han L., Qian X. (2012). Dalton Trans..

[cit13] Wu G., Li M., Zhu J., Chiu Lai K. W., Tong Q., Lu F. (2016). RSC Adv..

[cit14] Dhara A., Guchhait N., Mukherjee I., Mukherjee A., Bhattacharya S. C. (2016). RSC Adv..

[cit15] Manickam S., Lyer S. K. (2020). RSC Adv..

[cit16] Liu Y., Wang S.-Q., Zhao B.-X. (2015). RSC Adv..

[cit17] Li P., Zhou X., Huang R., Yang L., Tang X., Dou W., Zhao Q., Liu W. (2014). Dalton Trans..

[cit18] Varghese B., Al-Busafi S. N., Suliman F. O., Al-Kindy S. M. Z. (2017). RSC Adv..

[cit19] Tigreros A., Portilla J. (2020). RSC Adv..

[cit20] Wang S., Shao W., Li H., Liu C., Wang K., Zhang J. (2011). Eur. J. Med. Chem..

[cit21] De Sousa G. F., Garcia E., Gatto C. C., Resck I. S., Deflon V. M., Ardisson J. D. (2010). J. Mol. Struct..

[cit22] Havrylyuk D., Heidary D. K., Sun Y., Parkin S., Glazer E. C. (2020). ACS Omega.

[cit23] Zhang Z., Wang F.-W., Wang S.-Q., Ge F., Zhao B.-X., Miao J.-Y. (2012). Org. Biomol. Chem..

[cit24] Zhang Y. P., Li X. F., Yang Y. S., Wang J. L., Zhao Y. C., Xue J. J. (2021). J. Fluoresc..

[cit25] Ciupa A., Mahon M. F., De Bank P. A., Caggiano L. (2012). Org. Biomol. Chem..

[cit26] Claisen L., Claparède A. (1881). Ber. Dtsch. Chem. Ges..

[cit27] Ciupa A., De Bank P. A., Mahon M. F., Wood P. J., Caggiano L. (2013). MedChemComm.

[cit28] Hofmann S., Linden M., Neuner J., Weber F. N., Waldvogel S. R. (2023). Org. Biomol. Chem..

[cit29] Ding Y., Zhang T., Chen Q.-Y., Zhu C. (2016). Org. Lett..

[cit30] Landge S. M., Schmidt A., Outerbridge V., Török B. (2007). Synlett.

[cit31] Nguyen R., Huc I. (2003). Chem. Commun..

[cit32] Job P. (1928). Ann. Chim..

[cit33] Joshi B. P., Park J., Lee W. I., Lee K.-H. (2009). Talanta.

[cit34] Hu S., Zhang S., Gao C., Xu C., Gao Q. (2013). Spectrochim. Acta, Part A.

[cit35] Wei K., Deng Z., Lui Y., Kang M., Lui P., Yang X., Pai M., Zhang G. (2023). J. Photochem. Photobiol., A.

[cit36] Barceló-Oliver M., Terrón A., García-Raso A., Turel I., Morell M. (2010). Acta Crystallogr., Sect. E: Struct. Rep. Online.

[cit37] Singh U. P., Tyagi P., Pal S. (2009). Inorg. Chim. Acta.

[cit38] Asiri A. M., Al-Amari M. M., Khan S. A. (2020). J. Fluoresc..

[cit39] Zhang Y., Lui H., Gao W., Pu S. (2019). RSC Adv..

[cit40] Taş H., Adams J., Namyslo J. C., Schmidt A. (2021). RSC Adv..

[cit41] Shi Z., Tu Y., Pu S. (2018). RSC Adv..

[cit42] Feng E., Tu Y., Fan C., Liu G., Pu S. (2017). RSC Adv..

[cit43] Akula M., El-Khoury P. Z., Nag A., Bhattacharya A. (2014). RSC Adv..

[cit44] Lohar S., Pal S., Mukherjee M., Maji A., Demitri N., Chattopadhyay P. (2017). RSC Adv..

[cit45] Qu W.-J., Guan J., Wei T.-B., Yan G.-T., Lin Q., Zhang Y.-M. (2016). RSC Adv..

