# Supplementary material for: Novel pyrazoline and pyrazole “turn on” fluorescent sensors selective for Zn2+/Cd2+ at λem 480 nm and Fe3+/Fe2+ at λem 465 nm in MeCN
Source: RSC Adv. 2024 Jan 22;14(5):3519–24. doi: 10.1039/d4ra00036f (PMC10802262; doi:10.1039/d4ra00036f)

Electronic Supplementary Material (ESI) for RSC Advances.
This journal is © The Royal Society of Chemistry 2024

Electronic Supplementary Information †

Novel pyrazoline and pyrazole "turn on" fluorescent sensors selective for Zn^{2+} / Cd^{2+} at λ_{em}480 nm and Fe^{3+} / Fe^{2+} at λ_{em}465 nm in MeCN

Alexander Ciupa*

General Experimental **S1** .... Page 2

Synthetic Procedure **S2** .... Page 3

NMR Spectra of Compounds **S3** .... Page 12

Absorbance Spectra and Extinction Coefficients **S4** .... Page 22

Job Plot Analysis **S5** .... Page 24

Fluorescence Spectra **S6** .... Page 26

Limit of Detection Calculations **S7** .... Page 30

General Experimental (S1)

Chemicals, solvents and reagents were purchased from commercial sources and used without further purification. PE refers to petroleum ether, bp 40-60 °C. Spectroscopy was performed with CHROMASOLV® gradient grade acetonitrile for HPLC, ≥99.9%, from Sigma-Aldrich.

The metal complexes used in this study were: LiCl, NaCl, KCl, $CaCl_2$, $CuCl_2$, $NiCl_2$, $ZnCl_2$, $CdCl_2$, $RuCl_3$, $CoCl_2$, $MnCl_2$, $PbCl_2$, $ZnCl_2$, $FeSO_4$ and $FeCl_3$.

TLCs were carried out on Merck Aluminium backed TLC plates Silica Gel 60 F254 and viewed using UV light of wavelength 254 nm. Merck Silica Gel (0.040-0.063 mm) was used for column chromatography. Compounds were loaded as an oil, CH_2Cl_2 solution or dry loaded by adsorption onto silica.

NMR spectra were obtained on a Bruker Avance III (400 MHz) spectrometer and processed via TopSpin® software. The chemical shifts are recorded in parts per million (ppm) with reference to tetramethylsilane. The coupling constants *J* are quoted to the nearest 0.5 Hz and are not corrected.

High resolution Mass spectroscopy was performed on Bruker Quadrupole Time-of-Flight (qToF) mass spectrometer.

UV/Vis spectroscopy was performed on an Agilent Cary5000 in quartz cuvettes with a 1 cm pathlength using HPLC grade MeCN, 250-500 nm range with 0.2 sec dwell time. Detector switchover occurred at 350 nm.

FTIR spectroscopy was performed on a Bruker VERTEX 70 spectrometer.

Fluorescence spectroscopy was performed on an Edinburgh Instruments FLS1000 with a xenon excitation source, 5 nm bandwidths for both excitation and emission monochromator, scan speed of 1 nm and dwell time of 0.2 sec. Fluorescence quartz cuvettes with a 1 cm pathlength were used throughout with HPLC grade MeCN.

A 100 Watt 365 nm Analytikjena High intensity UV lamp or 254 nm 6 Watt Analytikjena TLC lamp was used for images of samples in cuvettes.

All figures were plotted using SigmaPlot® 14.5 software.

Synthetic Procedures (S2)

Synthesis of (E)-1-(6-acetylpyridin-2-yl)-3-phenylprop-2-en-1-one (**1**)

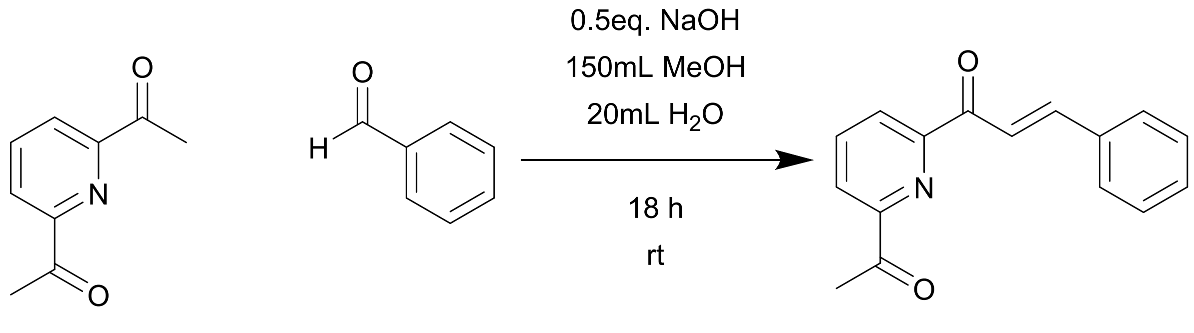

Benzaldehyde (0.318 g, 3 mmol) was added to a stirred solution of 2,6-Diacetylpyridine (0.978 g, 6 mmol) in 150 mL MeOH until fully dissolved. Sodium hydroxide (0.12 g, 3 mmol) dissolved in 20 mL water was added slowly over the course of 1 min and then left to stir at room temperature for 18 hours. After 18 hours the precipitate (unwanted bis-chalcone (2E,2'E)-1,1'-(pyridine-2,6-diyl)bis(3-phenylprop-2-en-1-one) was removed via filtration, the solvent removed under reduced pressure and the resulting solid was dissolved in 100 mL ethyl acetate and washed with 3 x 50 mL water (pH 4) washes. The ethyl acetate layer was separated, dried with magnesium sulphate and then the solvent removed under reduced pressure to afford pale yellow solid which was further dried in a drying oven at 80 ^{o}C for 24 hours. After 24 hours the required mono chalcone **1** was recrystallised from hexane to afford a pale-yellow solid (0.548 g, 73%).

M.p 124 ^{o}C (hexane);

Vmax (Solid)/cm^{-1} 1694, 1355, 994 and 762;

^{1}H NMR δ_H (400 MHz; $CDCl_3$) 2.90 (3 H, s, CH_3), 7.48 (3 H, m, CH), 7.75 (2 H, m, CH), 8.04 (2 H, m, CH), 8.26 (1 H, dd, *J* = 7.8 and 1.2 Hz, CH), 8.38 (1 H, d, *J* = 16.0 Hz, CH) and 8.40 (1 H, dd, *J* = 7.8 and 1.2 Hz, CH);

^{13}C NMR δ_c (400 MHz; $CDCl_3$) 25.8 (CH_3), 120.3 (CH), 124.6 (CH), 126.1 (CH), 127.5 (CH), 128.8 (CH), 130.1 (CH), 135.0 (Cq), 138.0 (CH), 145.3 (CH), 152.6 (Cq), 153.3 (Cq), 188.6 (Cq) and 199.4 (Cq);

HRMS *m/z* (qToF) Found 274.0852(M+Na^{+}). $C_{16}H_{14}NO_2$ requires 274.0844

.

Synthesis of (E)-1-(6-acetylpyridin-2-yl)-3-(4-fluorophenyl)prop-2-en-1-one (**2**)

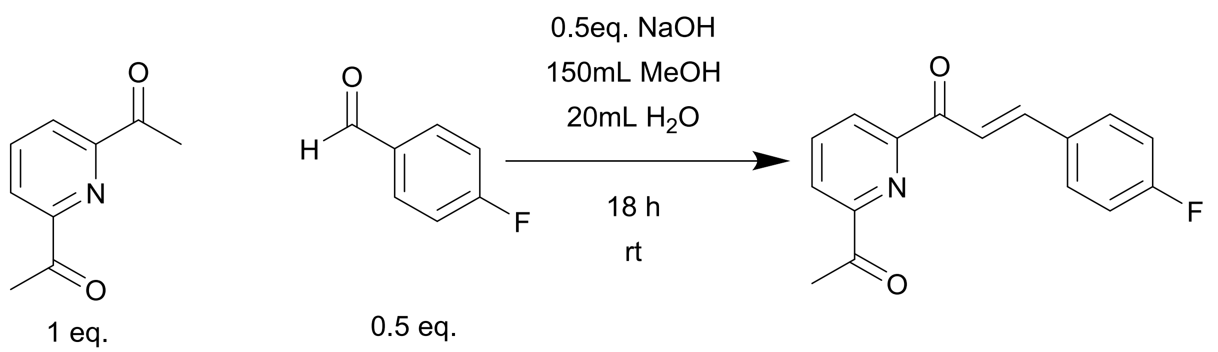

4-Fluorobenzaldehyde (0.728 g, 3mmol) was added to a stirred solution of 2,6-Diacetylpyridine (0.978 g, 6 mmol) in 150 mL MeOH until fully dissolved. Sodium hydroxide (0.12 g, 3 mmol) dissolved in 20 mL water was added slowly over the course of 1 min and then left to stir at room temperature for 18 hours. After 18 hours the precipitate (unwanted bis-chalcone 3-(4-fluorophenyl)-1-(6-((E)-3-(4-fluorophenyl)acryloyl)pyridin-2-yl)prop-2-en-1-one) was removed via filtration, the solvent removed under reduced pressure and the resulting solid was dissolved in 100 mL ethyl acetate and washed with 3 x 50 mL water (pH 4) washes. The ethyl acetate layer was separated, dried with magnesium sulphate and then the solvent removed under reduced pressure to afford pale yellow solid which was further dried in a drying oven at 80 ^{o}C for 24 hours. After 24 hours the required mono chalcone **2** was recrystallised from ethyl acetate to afford a pale-yellow solid (0.272 g, 34%).

M.p 150 ^{o}C (ethyl acetate);

Vmax (Solid)/cm^{-1} 1695, 1588, 1034 and 796;

^{1}H NMR δ_H (400 MHz; $CDCl_3$) 2.90 (3 H, s, CH_3), 7.17 (2 H, m, CH), 7.74 (2 H, m, CH), 7.99 (1 H, d, *J* = 16.0 Hz, CH), 8.06 (1 H, t, *J* = 7.8 Hz, CH), 8.27 (1H, dd, *J* = 7.7 and 1.1 Hz, CH), 8.30, (1H, d, *J* = 16 Hz, CH), 8.40 (1H, dd, J = 7.8 and 1.2 Hz, CH);

^{13}C NMR δ_c (400 MHz; $CDCl_3$) 25.8 (CH_3), 116.1 (CH), 120.0 (CH), 124.7 (CH), 126.2 (CH), 130.6 (CH), 131.2 (CH), 138.2 (CH), 144.0 (Cq), 152.6 (Cq), 153.3 (Cq), 188.4 (Cq), 199.4 (Cq);

HRMS *m/z* (qToF) Found 292.0832 (M+Na^+). $C_{16}H_{13}FNO_2$ requires 292.075.

Synthesis of (E)-1-(6-acetylpyridin-2-yl)-3-(4-methoxyphenyl)prop-2-en-1-one (**3**)

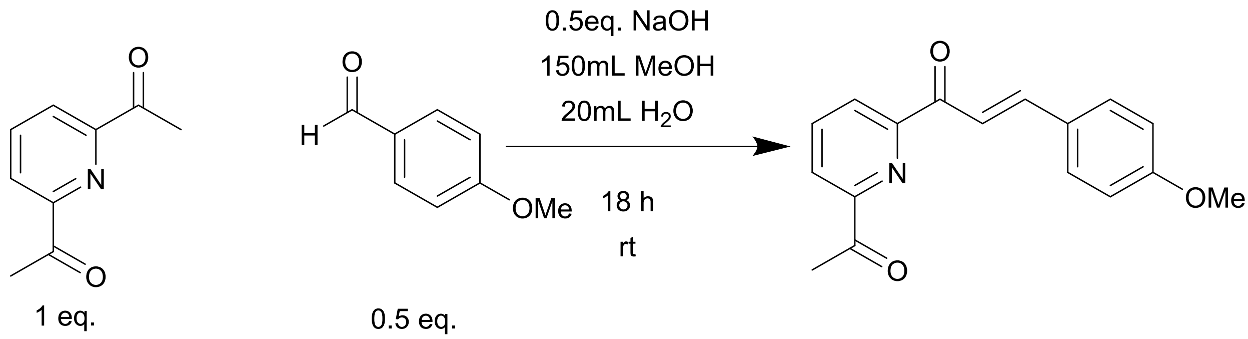

4-Methoxyobenzaldehyde (0.408 g, 3 mmol) was added to a stirred solution of 2,6-Diacetylpyridine (0.978 g, 6 mmol) in 150 mL MeOH until fully dissolved. Sodium hydroxide (0.12 g, 3 mmol) dissolved in 20 mL water was added slowly over the course of 1 min and then left to stir at room temperature for 18 hours. After 18 hours the precipitate (unwanted bis-chalcone(2E,2'E)-1,1'-(pyridine-2,6-diyl)bis(3-(4-methoxyphenyl)prop-2-en-1-one) was removed via filtration, the solvent removed under reduced pressure and the resulting solid was dissolved in 100 mL ethyl acetate and washed with 3 x 50 mL water (pH 4) washes. The ethyl acetate layer was separated, dried with magnesium sulphate and then the solvent removed under reduced pressure to afford pale yellow solid which was further dried in a drying oven at 80 °C for 24 hours. After 24 hours the required mono chalcone **3** was recrystallised from diethyl ether to afford a pale-yellow solid (0.320 g, 35%).

M.p 160 °C (diethyl ether)

Vmax (Solid)/cm^{-1} 1698, 1594, 1255, 992 and 834;

^{1}H NMR δ_H (400 MHz; $CDCl_3$) 2.90 (3 H, s, CH_3), 3.90 (3H,s, OCH_3), 7.00 (2 H, m, CH), 7.71 (2 H, m, CH), 8.00 (1 H, d, *J* = 16.0 Hz, CH), 8.05 (1 H, t, *J* = 7.72 Hz, CH), 8.26 (1 H, d, *J* = 16.0 Hz, CH), 8.25 (1 H, dd, *J* = 7.8 and 1.2 Hz, CH), 8.40 (1 H, dd, *J* = 7.8 and 1.2 Hz, CH);

^{13}C NMR δ_c (400 MHz; $CDCl_3$) 25.8 (CH_3), 55.5 (OCH_3), 114.5 (CH), 118.0 (CH), 124.4 (CH), 126.1 (CH), 127.8 (Cq), 130.6 (CH), 138.1 (CH), 145.1 (CH), 152.6 (Cq), 153.6 (Cq), 162.0 (Cq), 188.5 (Cq), 199.5 (Cq);

HRMS *m/z* (qToF) Found 304.0959 (M+Na+). $C_{17}H_{16}NO_3$ requires 304.095.

^{1}H NMR spectra consistent with:

E. C. Constable, E. Figgemeier, I. A. Hougen, C. E. Housecroft, M. Neuburger, S. Schaffner and L. A. Whall, *Dalton Trans.*, 2005, **7**, 1168.

Synthesis of 1-(6-(1-methyl-5-phenyl-4,5-dihydro-1H-pyrazol-3-yl)pyridin-2-yl)ethan-1-one (**4**)

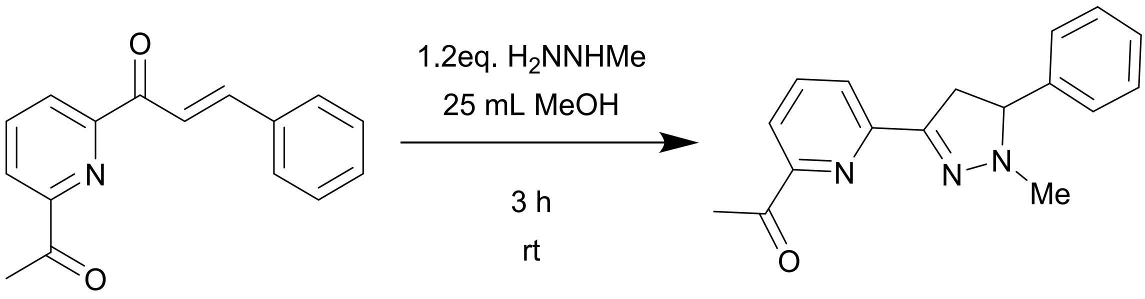

Methylhydrazine (0.6 mmol, 28 mg) was added to a stirred solution of (E)-1-(6-acetylpyridin-2-yl)-3-phenylprop-2-en-1-one (0.5 mmol, 0.126 g) in 25 mL MeOH slowly over the course of 2 min. The reaction mixture was allowed to stir for a further 3 h and then the solvent removed under reduce pressure to give a yellow oil. This was dissolved in 100 mL ethyl acetate and washed with 3 x 50 mL water (pH 4) washes, dried with magnesium sulphate and then the solvent reduced under reduced pressure to give a yellow pure which was further purified using column chromatography using silica gel and PE:EtOAc 9:1 to afford the desired **4** an a yellow oil (0.06 g, 43%).

Vmax (Solid)/cm^{-1} 1588, 1493, 1544, 811 and 695;

^{1}H NMR δ_H (400 MHz; $CDCl_3$) 2.69 (3 H, s, CH_3), 2.92 (3 H, s, CH_3), 3.13 (1 H, dd, *J* = 17.0 and 7.3, CH), 3.82 (1 H, dd, *J* = 17.1 and 10.5 Hz, CH), 4.27 (1 H, dd, *J* = 14.5 and 7.3 Hz, CH), 7.35 (1 H, m, CH), 7.41 (1 H, m, CH), 7.50 (1 H, m, CH), 7.81 (1 H, t, *J* = 7.8 Hz, CH), 7.93 (1 H, dd, *J* = 7.6 and 1.0 Hz, CH), 8.17 (1 H, dd, *J* = 8.0 and 1.0 Hz, CH);

^{13}C NMR δ_c (400 MHz; $CDCl_3$) 25.6 (CH_3), 40.9 (CH_3), 42.7 (CH_2), 73.5 (CH), 120.4 (CH), 123.6 (CH), 127.4 (CH), 127.9 (CH), 128.7 (CH), 136.7 (CH), 140.3 (Cq), 150.1 (Cq), 151.6 (Cq), 152.8 (Cq) and 200.3 (Cq);

HRMS *m/z* (qToF) Found 280.1446 (MH^+). $C_{17}H_{18}N_3O$ requires 280.145.

Synthesis of 1-(6-(5-(4-fluorophenyl)-1-methyl-4,5-dihydro-1H-pyrazol-3-yl)pyridin-2-yl)ethan-1-one (**5**)

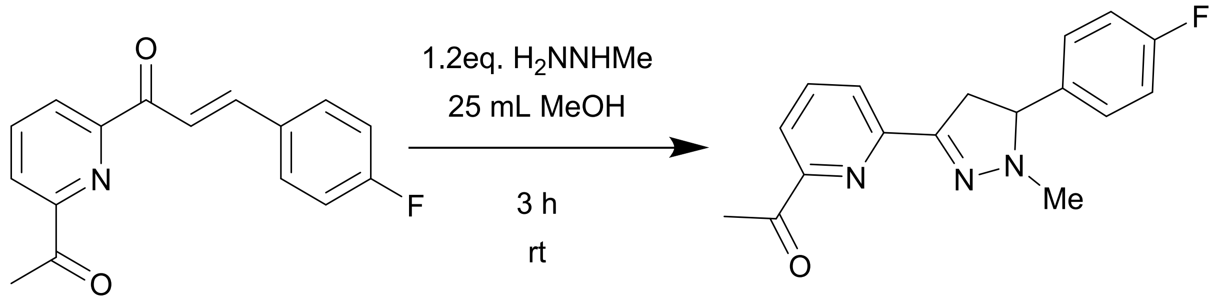

Methylhydrazine (0.6 mmol, 28 mg) was added to a stirred solution of ((E)-1-(6-acetylpyridin-2-yl)-3-(4-fluorophenyl)prop-2-en-1-one (0.5 mmol, 0.135 g) in 25 mL MeOH slowly over the course of 2 min. The reaction mixture was allowed to stir for a further 3 h and then the solvent removed under reduce pressure to give a yellow oil. This was dissolved in 100 mL ethyl acetate and washed with 3 x 50 mL water (pH 4) washes, dried with magnesium sulphate and then the solvent reduced under reduced pressure to give a yellow pure which was further purified using column chromatography using silica gel and PE:EtOAc 9:1 to afford the desired **5** as a yellow oil (0.06 g, 41%).

Vmax (Solid)/cm^{-1} 1689, 1513, 1197, 945 and 883;

^{1}H NMR δ_H (400 MHz; $CDCl_3$) 2.69 (3 H, s, CH_3), 2.90 (3 H, s, CH_3), 3.09 (1 H, dd, *J* = 17.1 and 14.5 Hz, CH), 3.81 (1 H, dd, *J* = 17.1 and 10.4 Hz, CH), 4.25 (1 H, dd, *J* = 14.5 and 10.4, CH), 7.10 (2 H, m, CH), 7.47 (2 H, m, CH), 7.82 (1 H, t, *J* = 7.8 Hz, CH), 7.94 (1 H, dd, *J* = 7.6 and 1.1 Hz, CH), 8.16 (1 H, dd, *J* = 8.0 and 1.2 Hz, CH);

^{13}C NMR δ_c (400 MHz; $CDCl_3$) 25.5 (CH_3), 41.0 (CH_3), 42.8 (CH_2), 72.8 (CH), 115.5 (CH), 115.7 (CH), 120.5 (CH), 123.6 (CH), 128.9 (CH), 135.9 (Cq), 136.7 (Cq), 150.1 (Cq), 151.5 (Cq), 152.3 (Cq), 200.2 (Cq);

HRMS *m/z* (qToF) Found 298.1356 (MH^+). $C_{17}H_{17}FN_3O$ requires 298.1332.

Synthesis of 1-(6-(5-(4-methoxyphenyl)-1-methyl-4,5-dihydro-1H-pyrazol-3-yl)pyridin-2-yl)ethan-1-one (**6**)

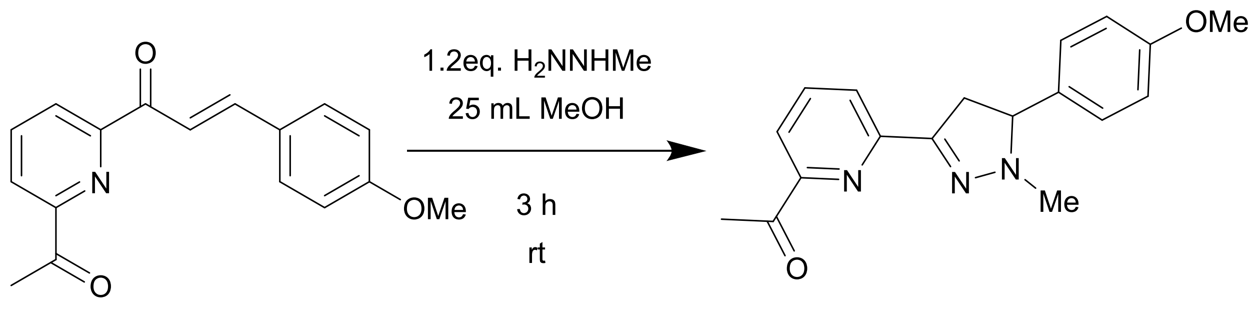

Methylhydrazine (0.38 mmol, 18 mg) was added to a stirred solution of (E)-1-(6-acetylpyridin-2-yl)-3-(4-methoxyphenyl)prop-2-en-1-one (0.32 mmol, 0.09 g) in 20 mL MeOH slowly over the course of 2 min. The reaction mixture was allowed to stir for a further 3 h and then the solvent removed under reduce pressure to give a yellow oil. This was dissolved in 100 mL ethyl acetate and washed with 3 x 50 mL water (pH 4) washes, dried with magnesium sulphate and then the solvent reduced under reduced pressure to give a yellow pure which was further purified using column chromatography using silica gel and PE:EtOAc 9:1 to afford the desired **6** as a yellow oil (0.036 g, 36%).

Vmax (Solid)/cm^{-1} 1690, 1377, 1041 and 883;

^{1}H NMR δ_H (400 MHz; $CDCl_3$) 2.61 (3 H, s, CH_3), 2.80 (3 H, s, CH_3), 3.01 (1 H, dd, J = 17.1 and 14.5 Hz, CH), 3.70 (1 H, dd, *J* = 17.1 and 10.4, CH), 3.76 (3 H, s, OCH_3), 4.13 (1 H, dd, *J* = 14.5 and 10.4, CH), 6.86 (2 H, m, CH), 7.33 (2 H, m, CH), 7.72 (1 H, t, *J* = 7.8 Hz, CH), 7.84 (1 H, dd, *J* = 7.6 and 1.1 Hz, CH), 8.07 (1 H, dd, *J* = 7.9 and 1.2 Hz, CH);

^{13}C NMR δ_c (400 MHz; $CDCl_3$) 25.5 (CH_3), 40.9 (CH_3), 42.5 (OCH_3), 55.3 (CH_2), 73.0 (CH), 114.1 (CH), 144.2 (CH), 120.3 (CH), 123.5 (CH), 128.6 (CH), 132.1 (Cq), 145.1 (Cq), 150.2 (Cq), 151.6 (Cq), 152.9 (Cq), 159.3 (Cq), 200.3 (Cq);

HRMS *m/z* (qToF) Found 310.1556 (MH^+). $C_{18}H_{20}N_3O_2$ requires 310.1523.

Synthesis of 1-(6-(1-methyl-5-phenyl-1H-pyrazol-3-yl)pyridin-2-yl)ethan-1-one (**7**)

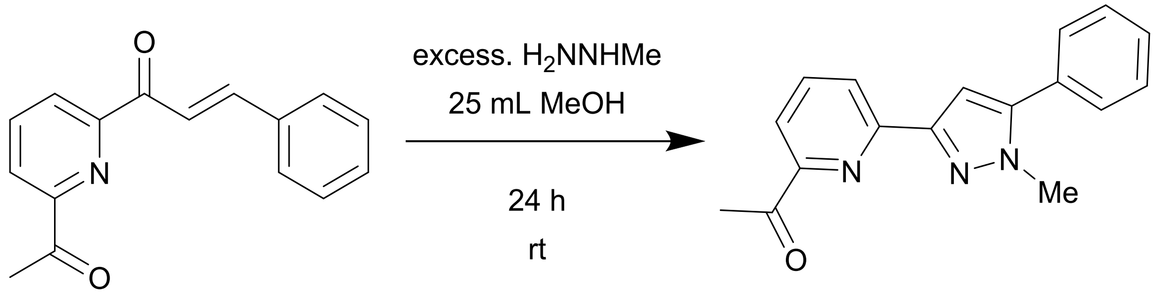

Methylhydrazine (6.4 mmol, 0.294 g) was added to a stirred solution of 1-(6-(1-methyl-5-phenyl-4,5-dihydro-1H-pyrazol-3-yl)pyridin-2-yl)ethan-1-one (0.8 mmol, 0.201 g) in 25 mL MeOH at room temperature and stirred continued for 24 hrs. After 24 h the solution was removed under reduced pressure to give a oil which was dissolved in 100 mL EtOAc and washed with 5 x 50 mL water (pH 4), the EtOAc layer separated and dried with magnesium sulphate then solvent reduced under reduced pressure to give a yellow pure which was further purified using column chromatography using silica gel and PE:EtOAc 9:1 to afford the desired **7** as a yellow oil (0.04 g, 18%).

Vmax (Solid)/cm^{-1} 2930, 1700, 1170, 856 and 776;

^{1}H NMR δ_H (400 MHz; $CDCl_3$) 2.82 (3 H, s, CH3), 4.00 (3 H, s, CH3), 7.07 (1 H, s, CH), 7.53 (5 H, m, CH), 7.88 (1 H, t, *J* = 7.8 Hz, CH), 7.97 (2 H, dd, *J* = 7.7 and 1.2 Hz, CH), 8.20 (1 H, dd, *J* = 7.7 and 1.2 Hz, CH);

^{13}C NMR δ_c (400 MHz; $CDCl_3$) 25.7 (CH_3), 37.8 (CH_3), 105.0 (CH), 120.0 (CH), 123.0 (CH), 125.1 (CH), 127.5 (CH), 128.6 (CH), 130.4 (Cq), 137.4 (CH), 145.4 (Cq), 150.3 (Cq), 151.7 (Cq), 153.2 (Cq), 200.6 (Cq);

HRMS *m/z* (qToF) Found 278.1293 (MH^+). $C_{17}H_{16}N_3O$ requires 278.1317.

Synthesis of 1-(6-(5-(4-fluorophenyl)-1-methyl-1H-pyrazol-3-yl)pyridin-2-yl)ethan-1-one (**8**)

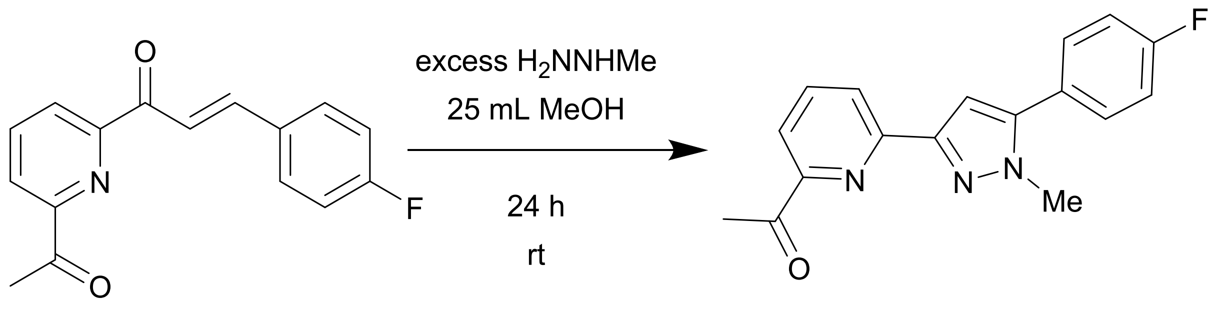

Methylhydrazine (4 mmol, 0.184 g) was added to a stirred solution of ((E)-1-(6-acetylpyridin-2-yl)-3-(4-fluorophenyl)prop-2-en-1-one (0.5 mmol, 0.135 g) in 25 mL MeOH at room temperature and stirred continued for 24 hr. After 24 h the solution was removed under reduced pressure to give a oil which was dissolved in 100 mL EtOAc and washed with 5 x 50 mL water (pH 4), the EtOAc layer separated and dried with magnesium sulphate then solvent reduced under reduced pressure to give a yellow pure which was further purified using column chromatography using silica gel and PE:EtOAc 9:1 to afford the desired **8** an a yellow oil (0.014 g, 9%).

Vmax (Solid)/cm^{-1} 2919, 1689, 1586, 1220 and 805;

^{1}H NMR δ_H (400 MHz; $CDCl_3$) 2.81 (3 H, s, CH_3), 3.97 (3 H, s, CH_3), 7.05 (1 H, s, CH), 7.22 (2 H, m, CH), 7.50, 2 H, m, CH), 7.89 (1 H, t, *J* = 7.8 Hz, CH), 7.98 (1 H, dd, *J* = 7.7 and 1.2 Hz, CH), 8.19, (1 H, dd, *J* = 7.7 and 1.2 Hz, CH);

^{13}C NMR δ_c (400 MHz; $CDCl_3$) 25.7 (CH_3), 37.8 (CH_3), 105.1 (CH), 115.8 (CH), 116.0 (CH), 120.2 (CH), 123.0 (CH), 130.6 (CH), 130.7 (Cq), 137.3 (Cq), 144.3 (Cq), 150.3 (Cq), 151.6 (Cq), 153.2 (Cq), 200.6 (Cq);

HRMS *m/z* (qToF) Found 296.1199 (MH^+). $C_{17}H_{15}FN_3O$ requires 296.1195.

Synthesis of 1-(6-(5-(4-methoxyphenyl)-1-methyl-1H-pyrazol-3-yl)pyridin-2-yl)ethan-1-one (**9**)

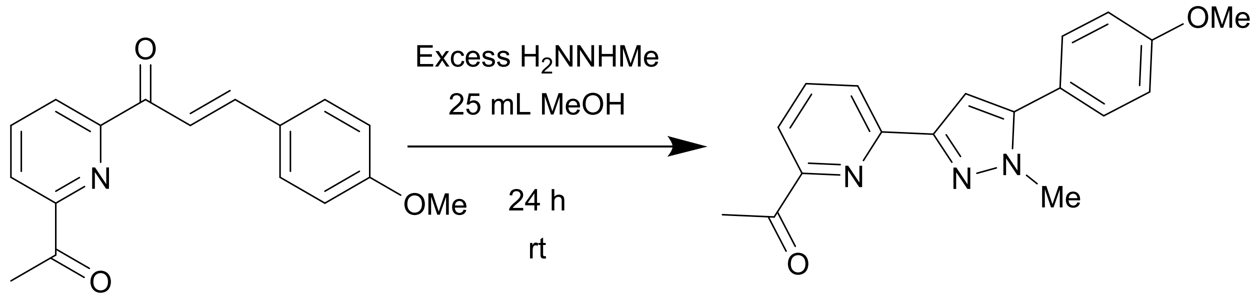

Methylhydrazine (4 mmol, 0.184 g) was added to a stirred solution of (E)-1-(6-acetylpyridin-2-yl)-3-(4-methoxyphenyl)prop-2-en-1-one (0.5 mmol, 0.135 g) in 25 mL MeOH at room temperature and stirred continued for 24 h. After 24 h the solution was removed under reduced pressure to give an oil which was dissolved in 100 mL EtOAc and washed with 5 x 50 mL water (pH 4), the EtOAc layer separated and dried with magnesium sulphate then solvent reduced under reduced pressure to give a yellow pure which was further purified using column chromatography using silica gel and PE:EtOAc 9:1 to afford the desired **9** as a yellow oil (0.013 g, 8%).

Vmax (Solid)/cm^{-1} 2973, 1690, 1377, 1046 and 880;

^{1}H NMR δ_H (400 MHz; $CDCl_3$) 2.71 (3 H, s, CH_3), 3.79 (3 H, s, CH_3), 4.30 (3 H, s OCH_3), 6.82 (1 H, s, CH), 6.89 (2 H, m, CH), 7.70 (2 H, m, CH), 7.77 (2 H, dd, *J* = 7.8 and 1.2 Hz, CH), 7.85 (1 H, t, *J* = 7.8 Hz, CH), 7.93 (1 H, dd, *J* = 7.7 and 1.2 Hz, CH);

^{13}C NMR δ_c (400 MHz; $CDCl_3$) 26.1 (CH_3), 40.0 (CH_3), 55.3 (OCH_3), 103.4 (CH), 109.2 (CH), 109.5 (CH), 114.1 (CH), 120.1 (CH), 125.8 (CH), 126.8 (Cq), 131.4 (Cq), 132.3 (Cq) 137.8 (Cq), 150.6 (Cq) 158.1 (Cq), 199.6 (Cq);

HRMS *m/z* (qToF) Found 308.1399 (MH^+). $C_{18}H_{18}N_3O_2$ requires 308.1381.

NMR Spectra (S3)

(E)-1-(6-acetylpyridin-2-yl)-3-phenylprop-2-en-1-one – Chalcone (1)

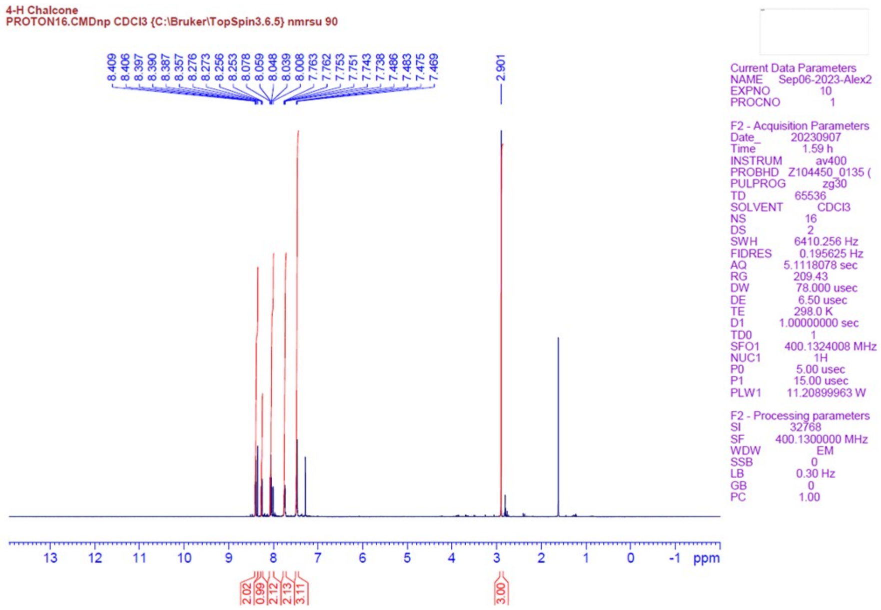

^{1}H NMR, $CDCl_3$, 400 MHz.

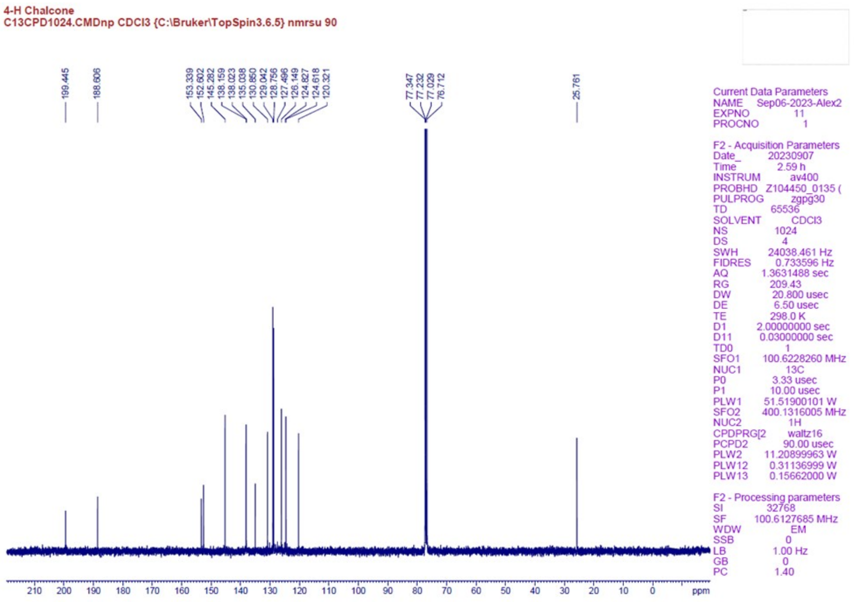

^{13}C NMR, $CDCl_3$, 400 MHz.

(E)-1-(6-acetylpyridin-2-yl)-3-(4-fluorophenyl)prop-2-en-1-one – Chalcone (2)

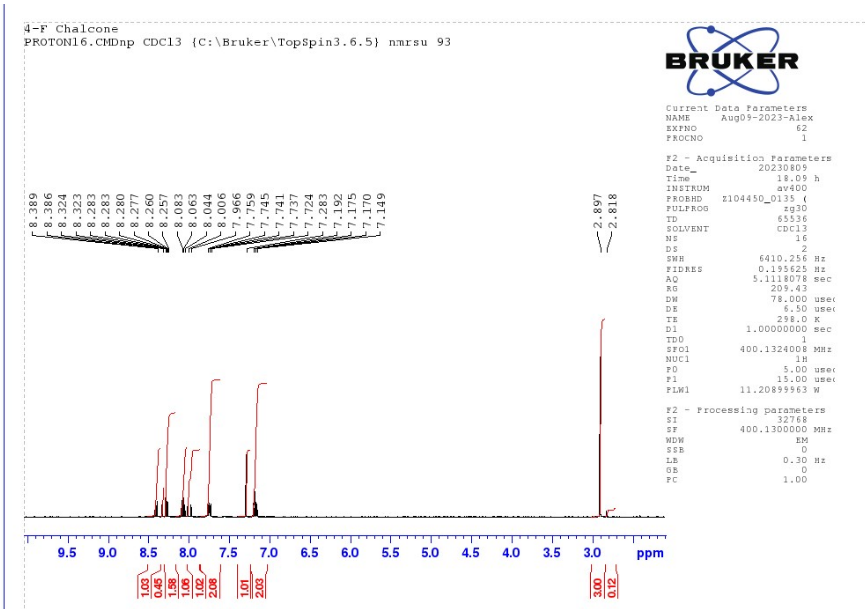

^{1}H NMR, $CDCl_3$, 400 MHz.

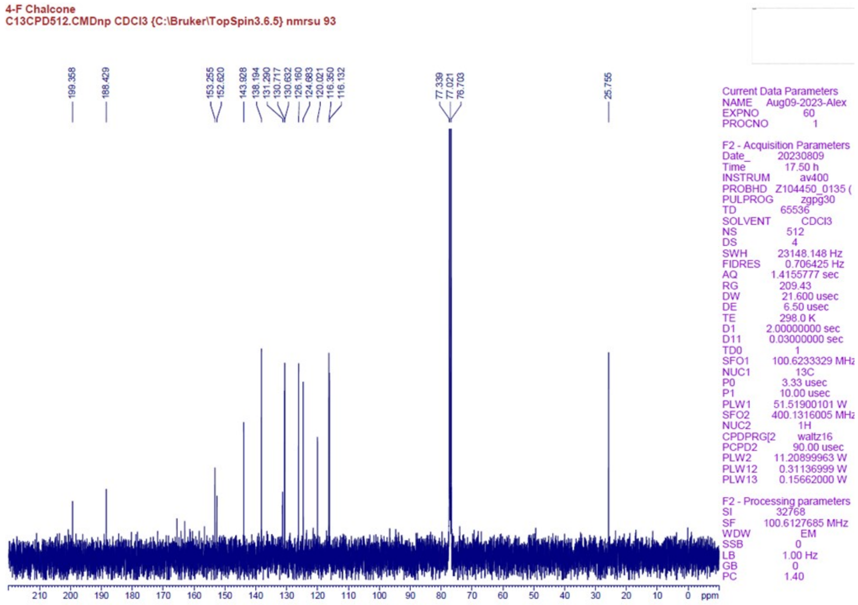

^{13}C NMR, $CDCl_3$, 400 MHz.

(E)-1-(6-acetylpyridin-2-yl)-3-(4-methoxyphenyl)prop-2-en-1-one – Chalcone (3)

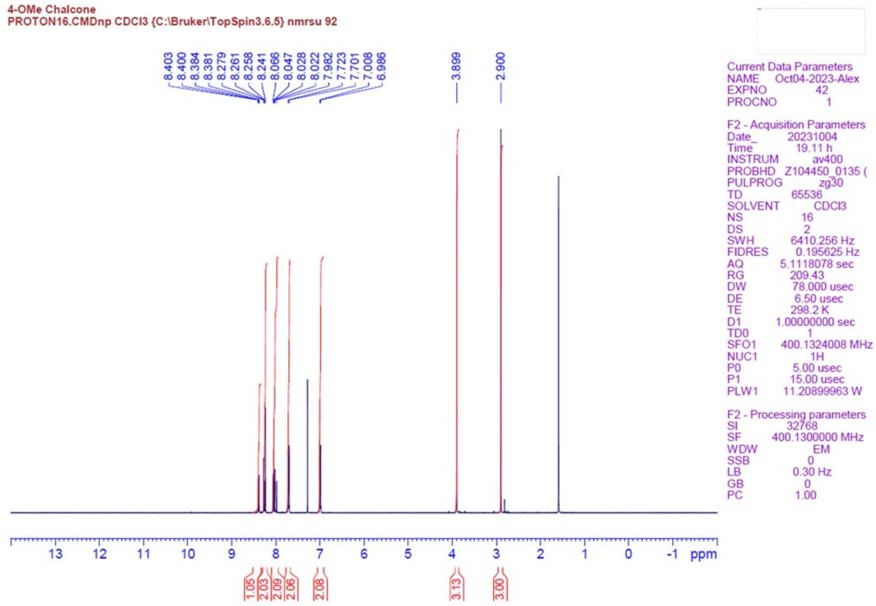

^1H NMR, $CDCl_3$, 400 MHz.

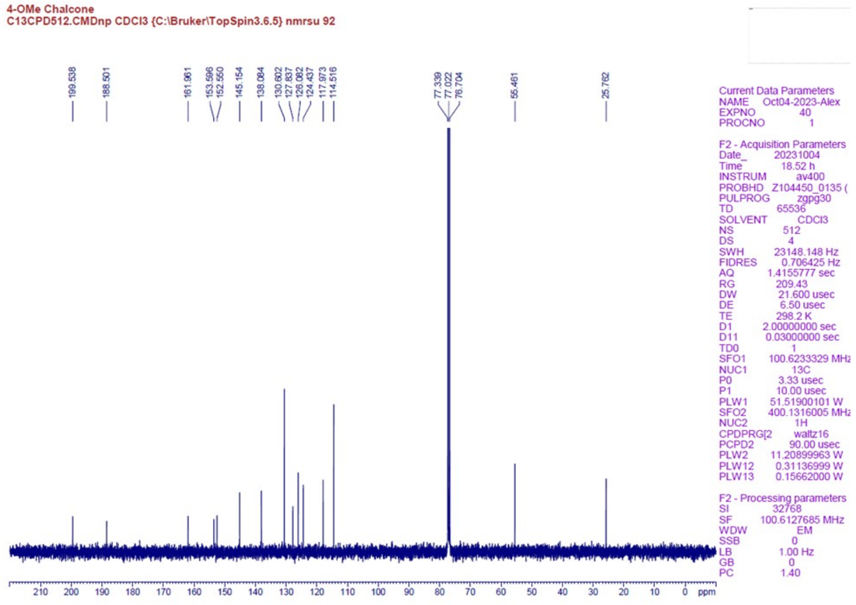

^{13}C NMR, $CDCl_3$, 400 MHz.

1-(6-(1-methyl-5-phenyl-4,5-dihydro-1H-pyrazol-3-yl)pyridin-2-yl)ethan-1-one – Pyrazoline (4)

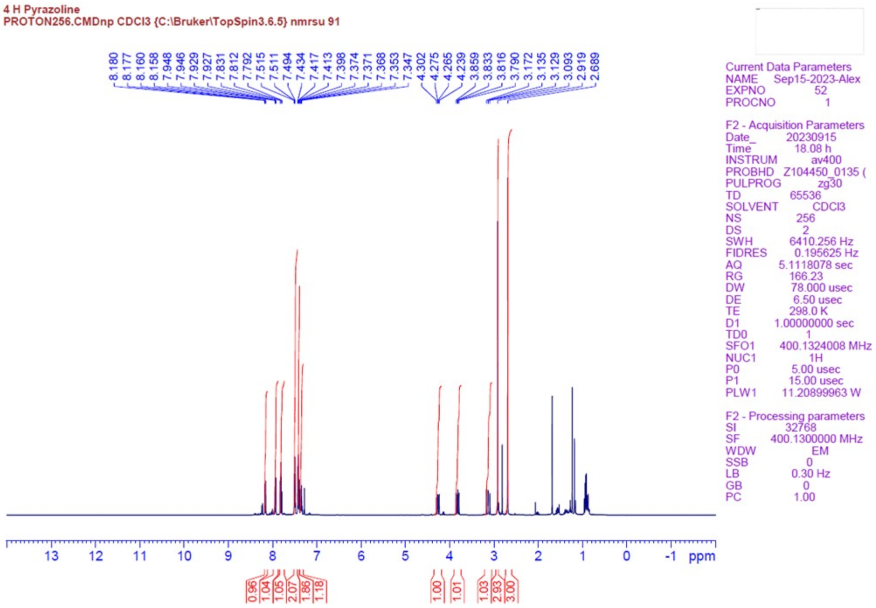

^{1}H NMR, $CDCl_3$, 400 MHz.

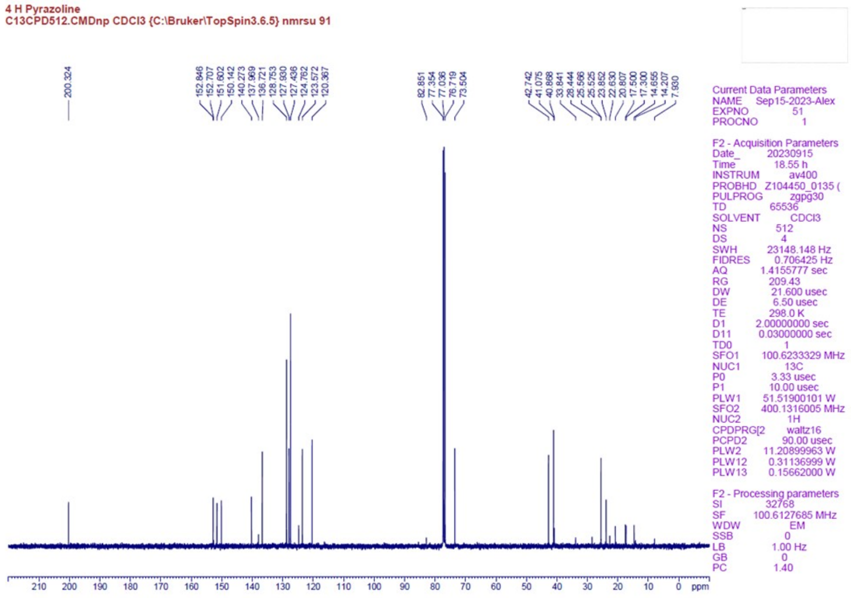

^{13}C NMR, $CDCl_3$, 400 MHz.

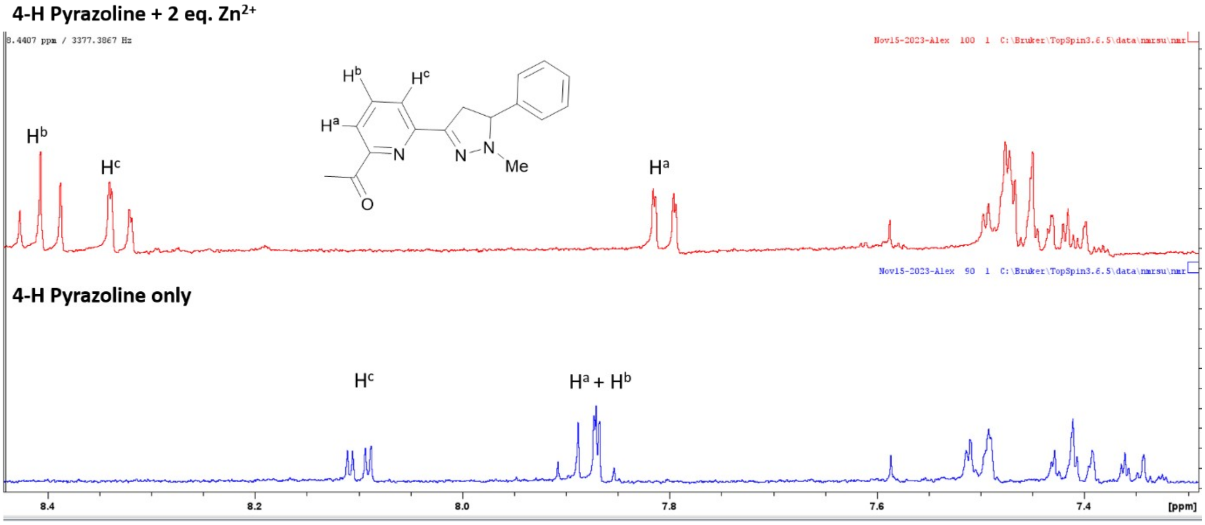

^{1}H NMR with and without Zn^{2}, MeCN-d_3, 400 MHz.

1-(6-(5-(4-fluorophenyl)-1-methyl-4,5-dihydro-1H-pyrazol-3-yl)pyridin-2-yl)ethan-1-one – Pyrazoline (5)

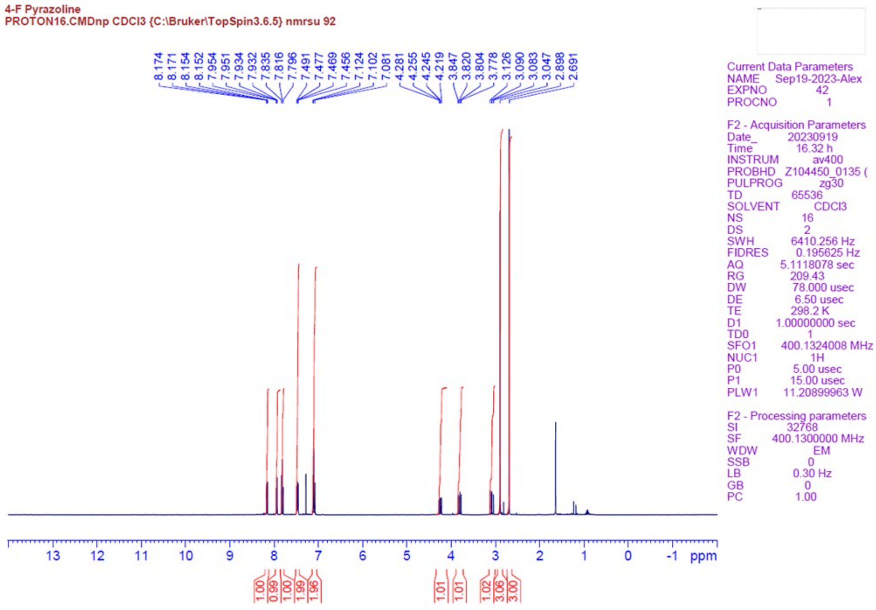

^{1}H NMR, $CDCl_3$, 400 MHz.

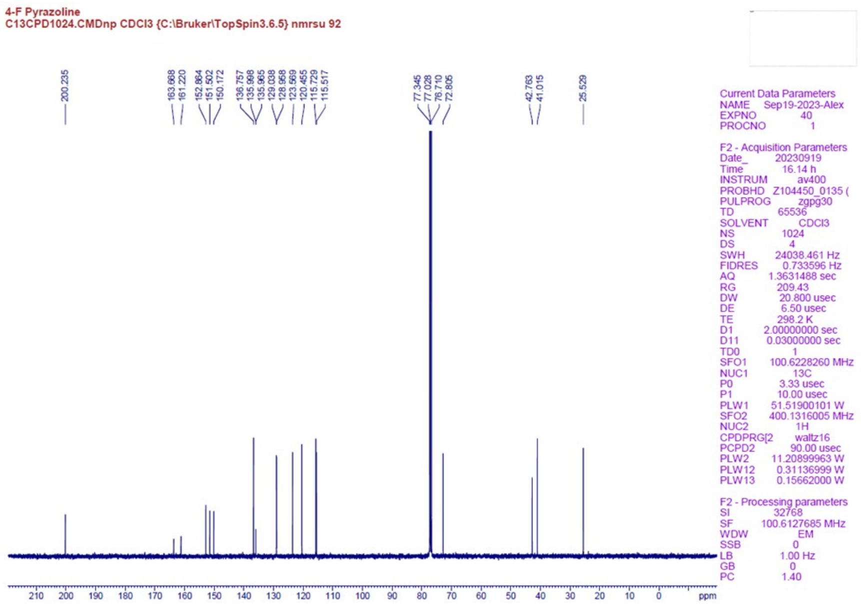

^{13}C NMR, $CDCl_3$, 400 MHz.

4-F Pyrazoline + 2 eq. Zn^{2+}

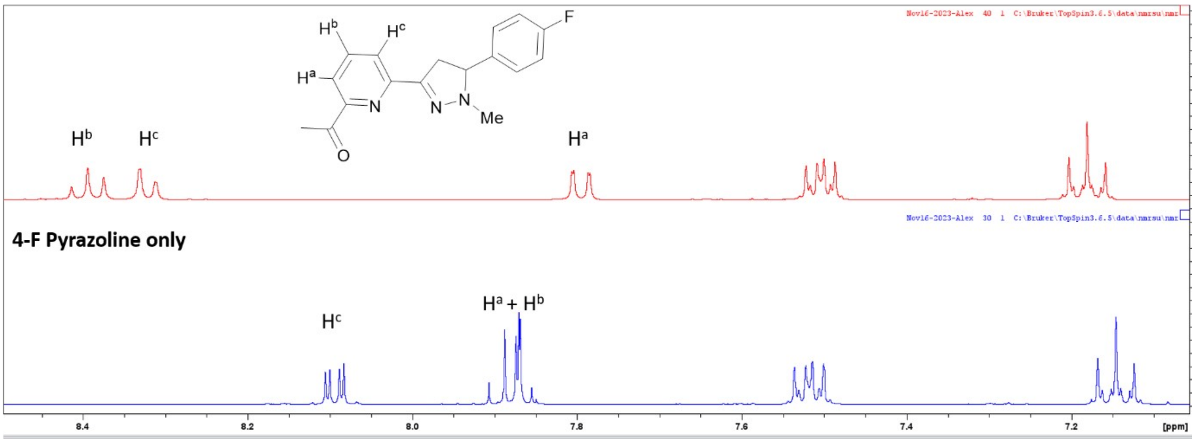

^{1}H NMR with and without Zn^{2+}, MeCN-d_3, 400 MHz.

1-(6-(5-(4-methoxyphenyl)-1-methyl-4,5-dihydro-1H-pyrazol-3-yl)pyridin-2-yl)ethan-1-one – Pyrazoline (6)

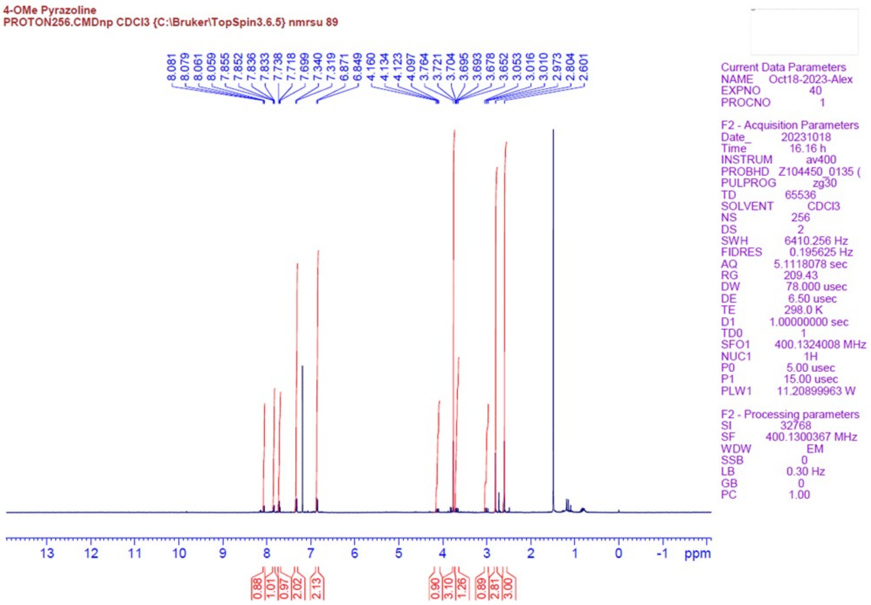

^{1}H NMR, $CDCl_3$, 400 MHz.

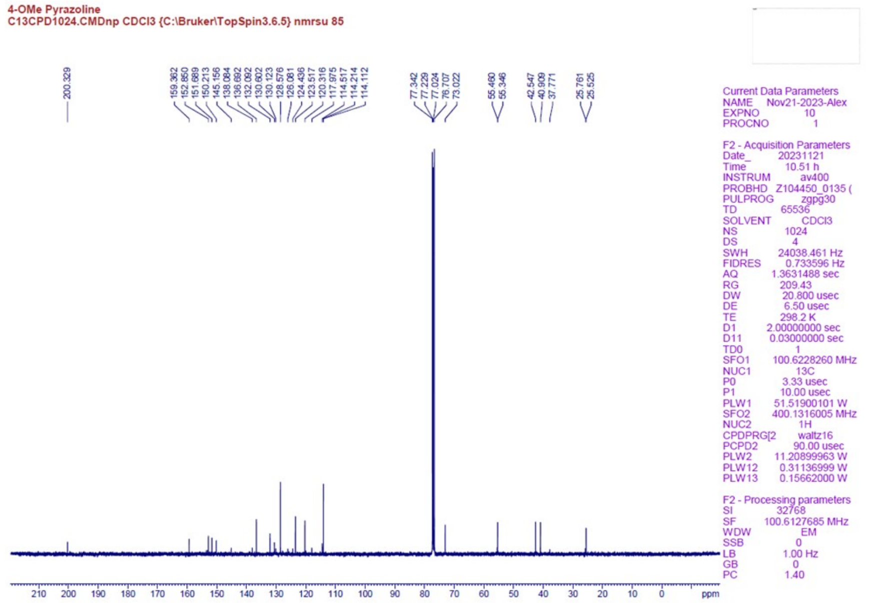

^{13}C NMR, $CDCl_3$, 400 MHz.

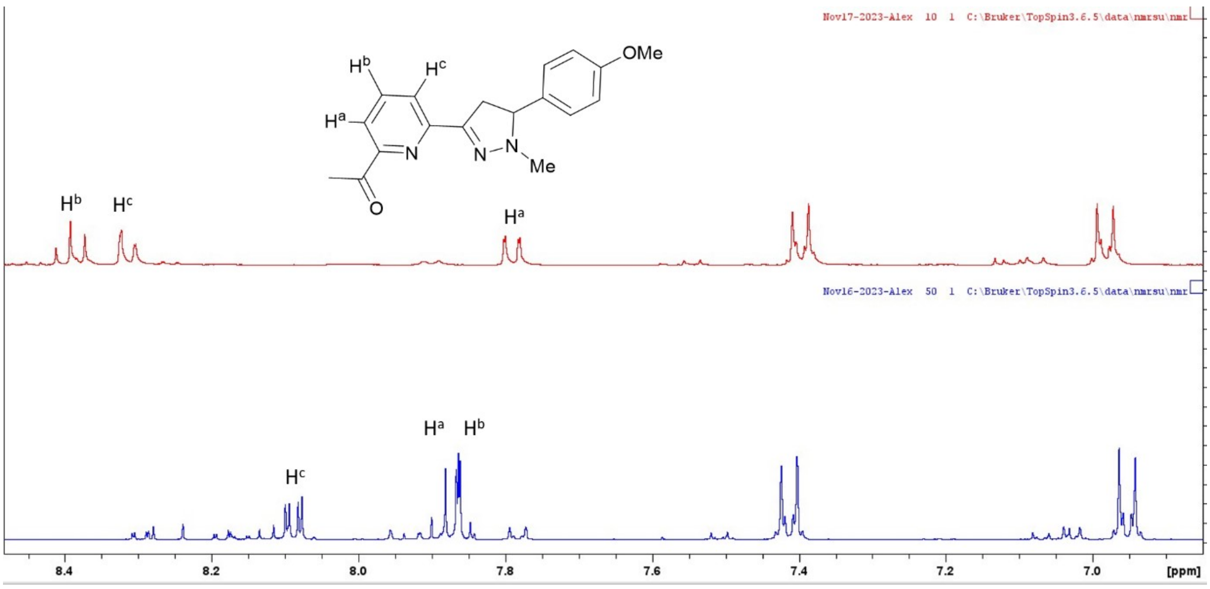

^{1}H NMR with and without Zn^{2+}, MeCN-d_3, 400 MHz.

1-(6-(1-methyl-5-phenyl-1H-pyrazol-3-yl)pyridin-2-yl)ethan-1-one – Pyrazole (7)

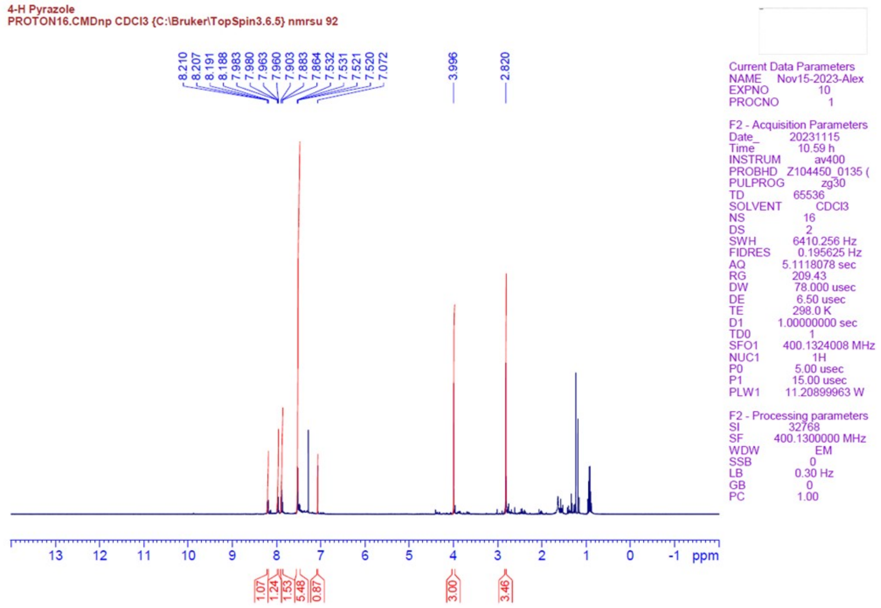

^{1}H NMR, $CDCl_3$, 400 MHz.

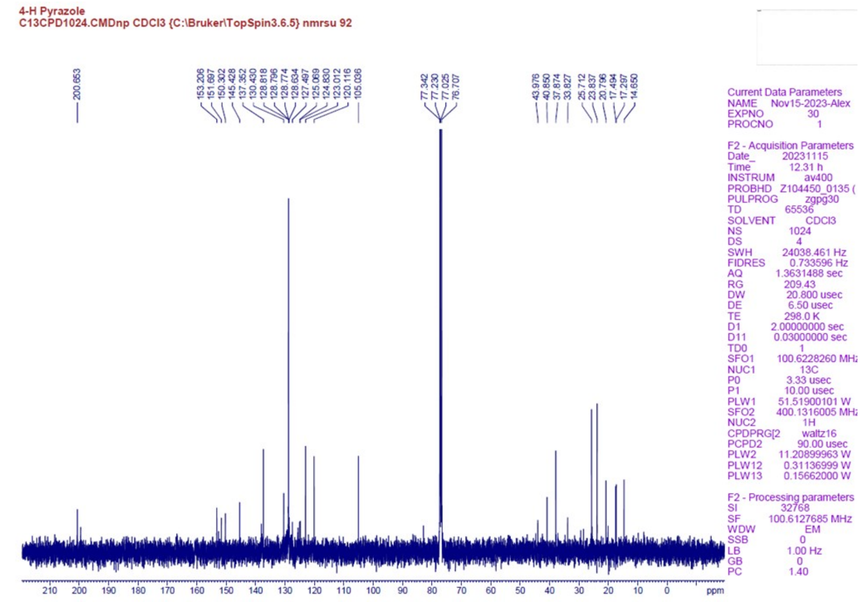

^{13}C NMR, , $CDCl_3$, 400 MHz.

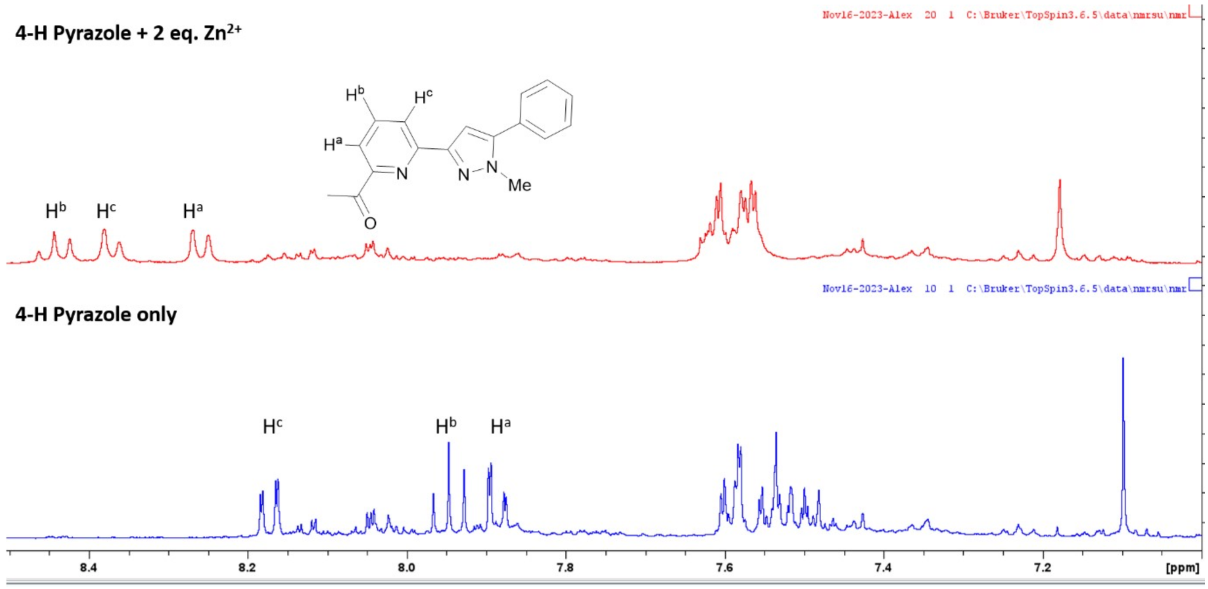

^{1}H NMR with and without Zn^{2++}, MeCN-d_3, 400 MHz.

1-(6-(5-(4-fluorophenyl)-1-methyl-1H-pyrazol-3-yl)pyridin-2-yl)ethan-1-one – Pyrazole (8)

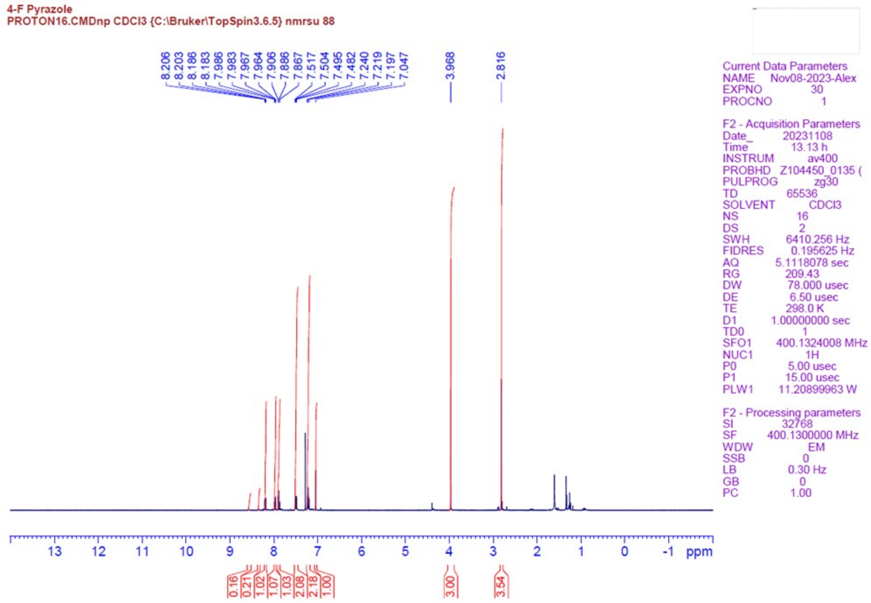

^{1}H NMR, , $CDCl_3$, 400 MHz.

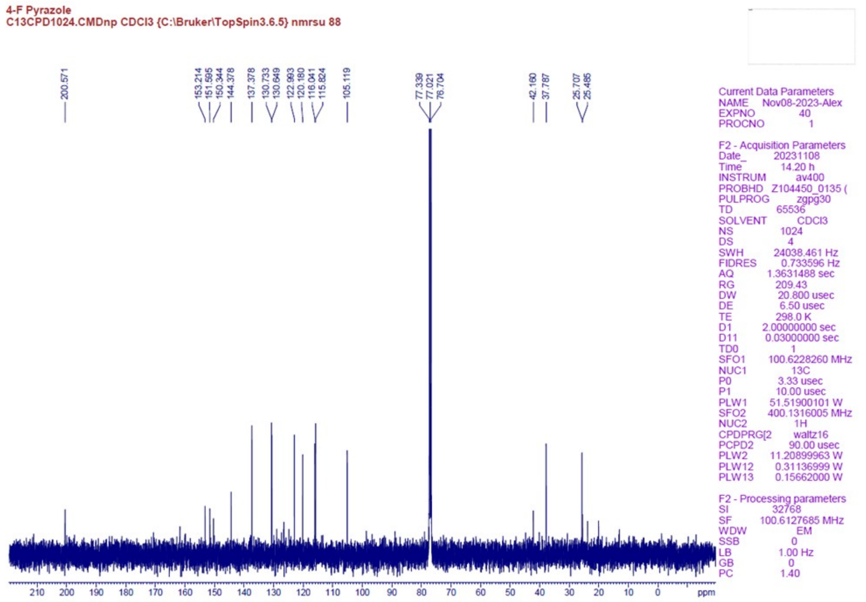

^{13}C NMR, , $CDCl_3$, 400 MHz.

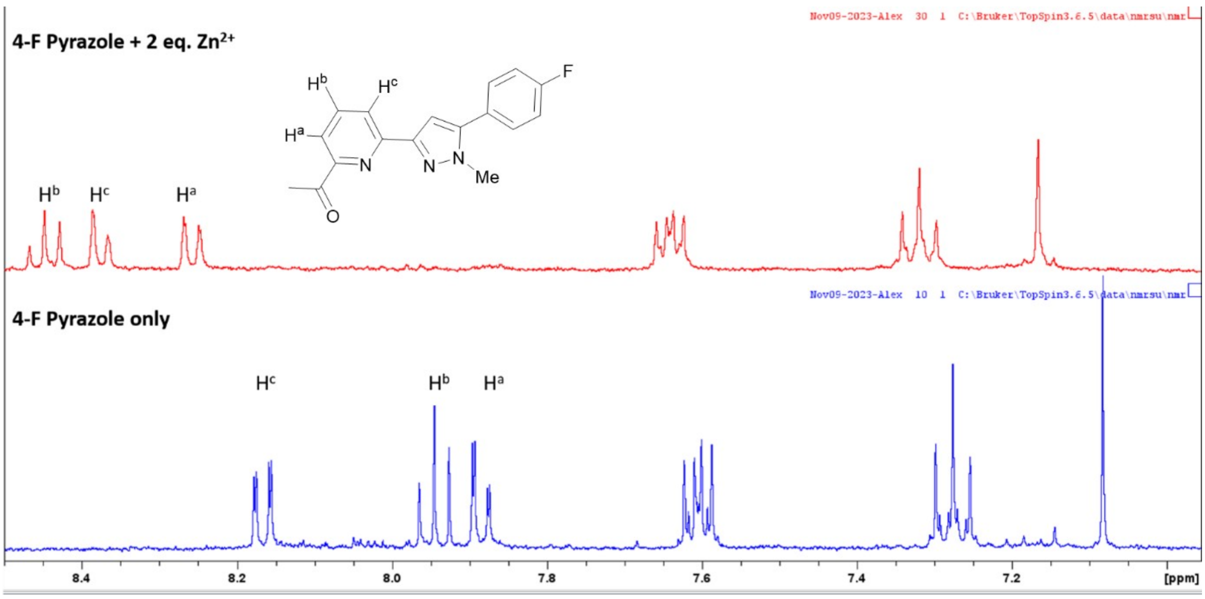

^{1}H NMR with and without Zn^{2+}, MeCN-d_3, 400 MHz.

1-(6-(5-(4-methoxyphenyl)-1-methyl-1H-pyrazol-3-yl)pyridin-2-yl)ethan-1-one – Pyrazole (9)

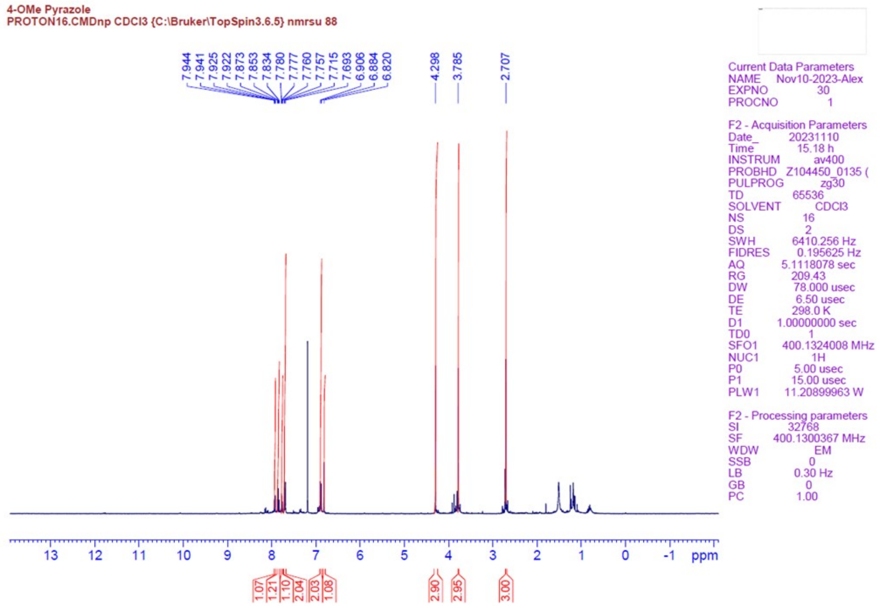

^{1}H NMR, $CDCl_3$, 400 MHz.

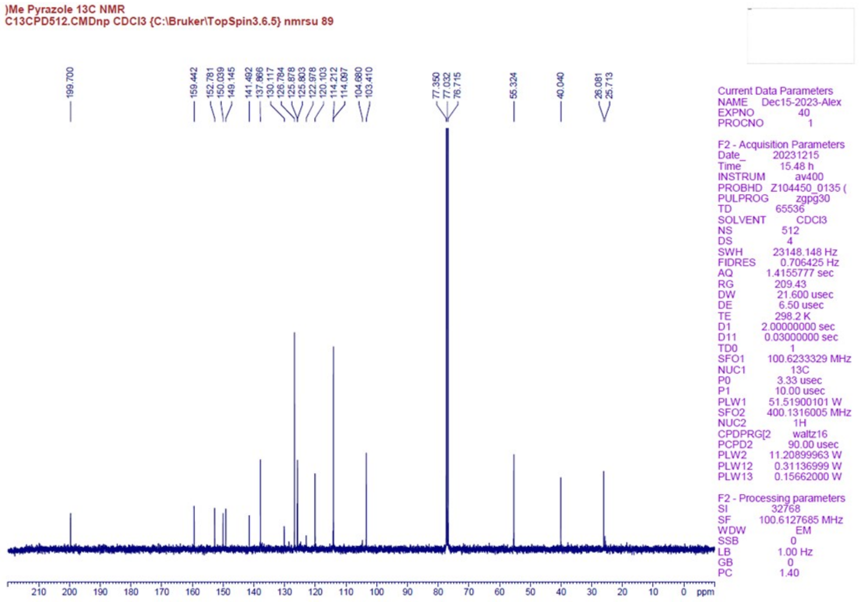

^{13}C NMR, $CDCl_3$, 400 MHz.

4-H Bis Chalcone

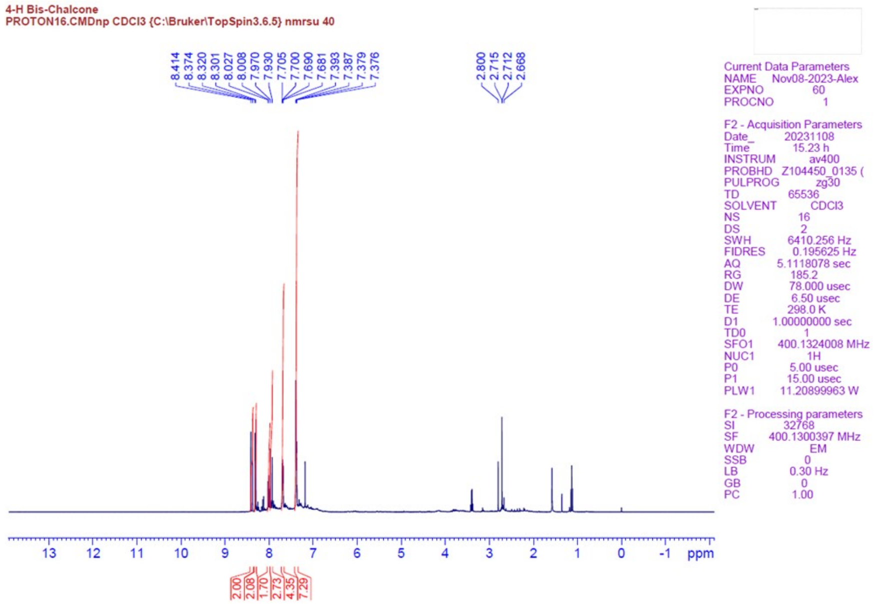

^{1}H NMR, $CDCl_3$, 400 MHz.

4-OMe Bis-chalcone

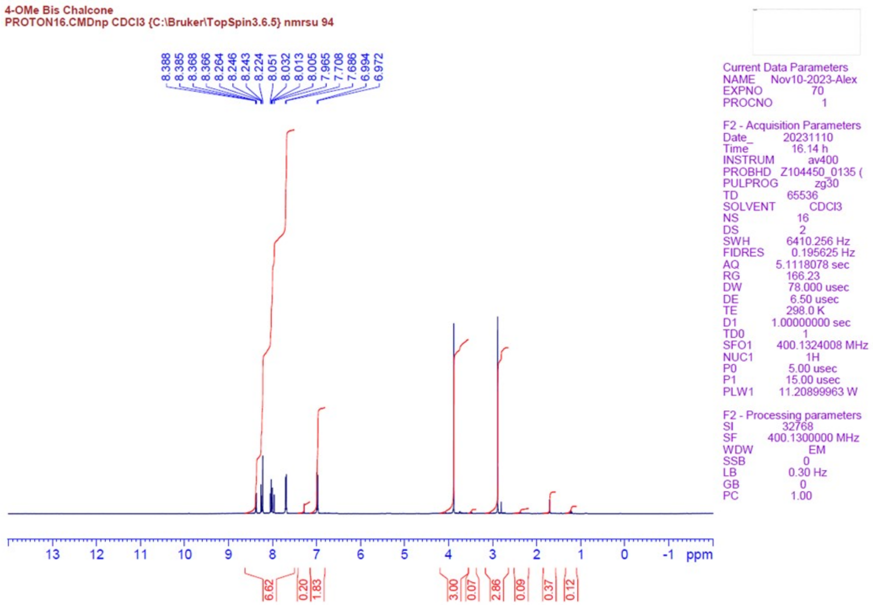

^1H NMR, $CDCl_3$, 400 MHz.

Data is consistent with previous bis-chalcones in the literature;

(a) E. C. Constable, E. Figgemeier, I. A. Hougen, C. E. Housecroft, M. Neuburger, S. Schaffnerand L. A. Whall, *Dalton Trans*., 2005, **7**, 1168;

(b) L. Sansalone, E.A. Veliz, N. G. Myrthil, V. Stathias, W. Walters, I. I. Torrens, S. C. Schürer, S. Vanni, R. M. Leblanc, R. M. Graham, *Cancers,* 2019, **11**, 357.

Absorbance Spectra and Extinction Coefficients (S4)

Pyrazoline (4)

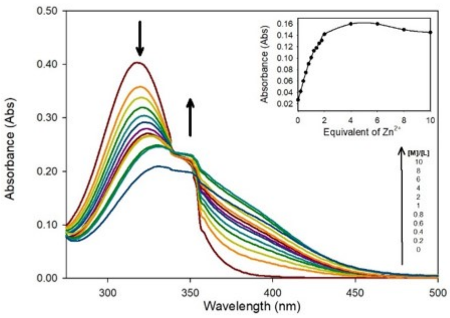

(4) only, Abs @ 320 nm = 0.331, Ɛ = 16550 M^{-1} cm^{-1}

(4) + 6 eq. Zn^{2+}, Abs @336 nm =0.210, Ɛ = 10500 M^{-1} cm^{-1}

Pyrazoline (5)

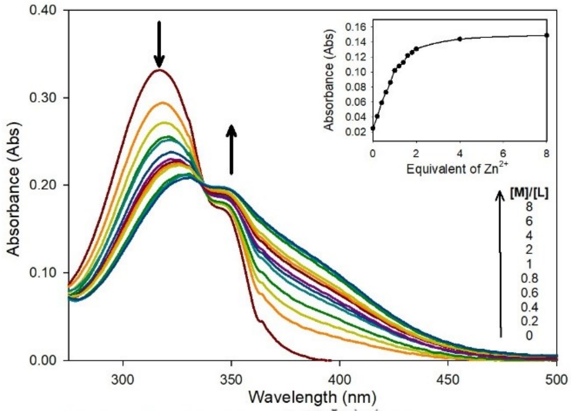

(5) only, Abs @ 320 nm = 0.403, Ɛ = 20150 M^{-1} cm^{-1}

(5) + 6 eq. Zn^{2+}, Abs @336 nm =0.245, Ɛ = 12250 M^{-1} cm^{-1}

Pyrazoline (6)

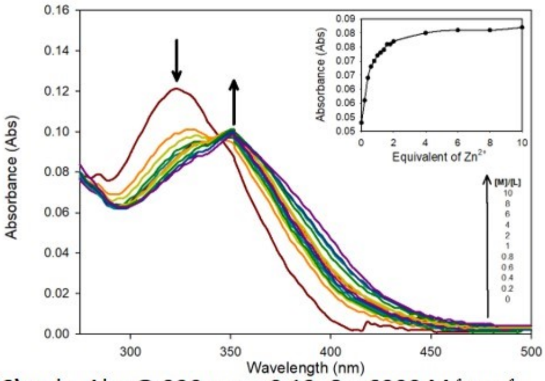

(6) only, Abs @ 320 nm = 0.12, Ɛ = 6000 M^{-1} cm^{-1}

(6) + 6 eq. Zn^{2+}, Abs @336 nm =0.113, Ɛ = 5650 M^{-1} cm^{-1}

Pyrazole (7)

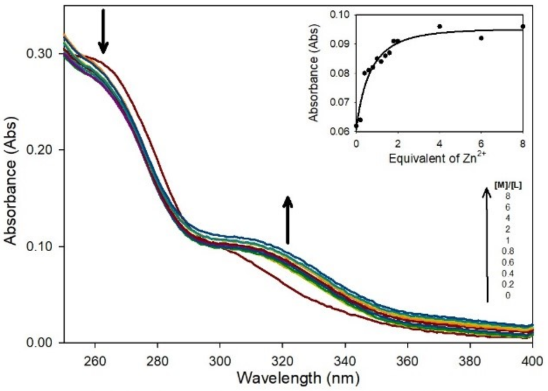

(7) Only, Abs @ 270 nm = 0.26 , $\mathcal{E}$ = 13,000 M^{-1} cm^{-1}
(7) + 6 eq. Zn^{2+} Abs @ 320nm = 0.091, $\mathcal{E}$ = 4,550 M^{-1} cm^{-1}

Pyrazole (8)

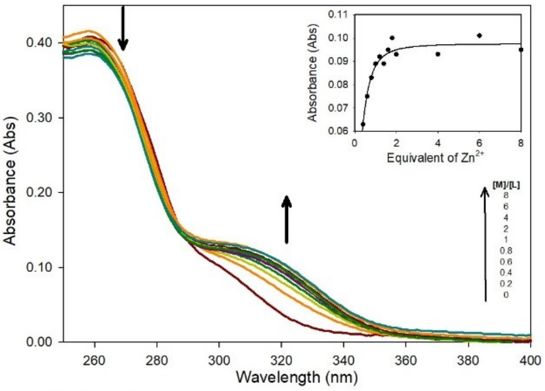

(8) Only, Abs @ 270 nm = 0.358 , $\mathcal{E}$ = 17,900 M^{-1} cm^{-1}
(8) + 6 eq. Zn^{2+} Abs @ 320nm = 0.106 , $\mathcal{E}$ = 5,300 M^{-1} cm^{-1}

Pyrazole (9)

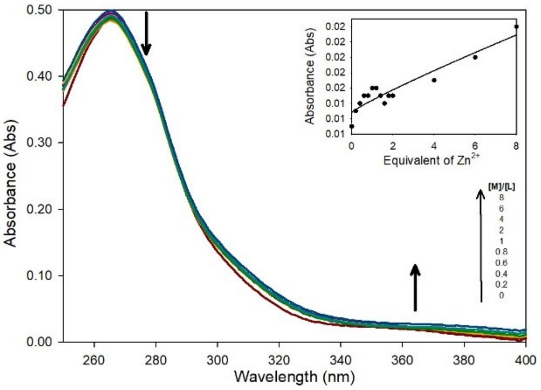

(9) Only, Abs @ 270 nm = 0.074 , $\mathcal{E}$ = 3,700 M^{-1} cm^{-1}
(9) + 6 eq. Zn^{2+} Abs @ 370nm = 0.082 , $\mathcal{E}$ = 4,100 M^{-1} cm^{-1}

Job Plot Analysis (S5)

Pyrazoline (4), [4] +[Zn^{2+}] = 100 µM, MeCN

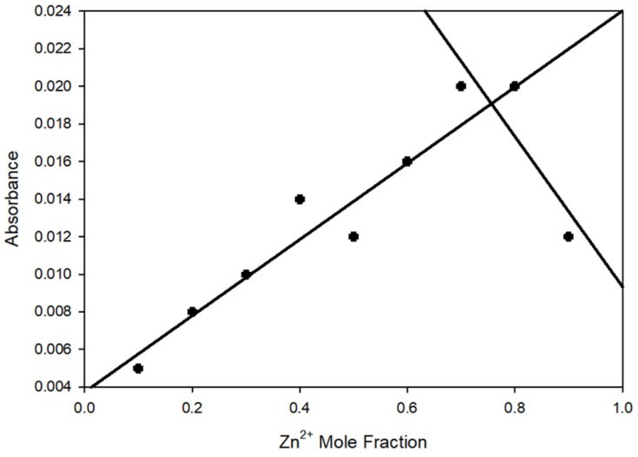

Pyrazoline (5), [5] +[Zn^{2+}] = 100 µM, MeCN

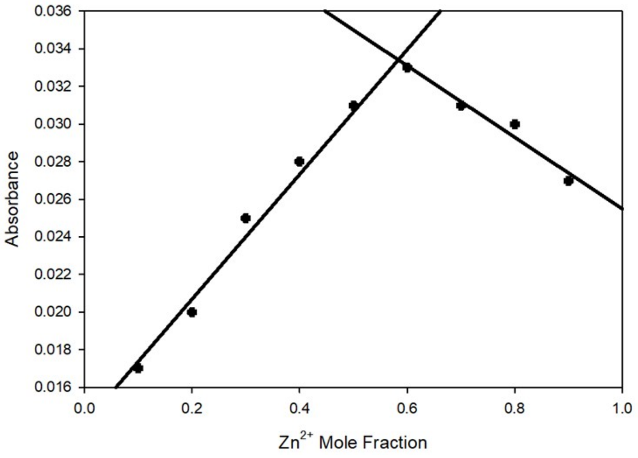

Pyrazoline (6), [6] +[Zn^{2+}] = 100 µM, MeCN

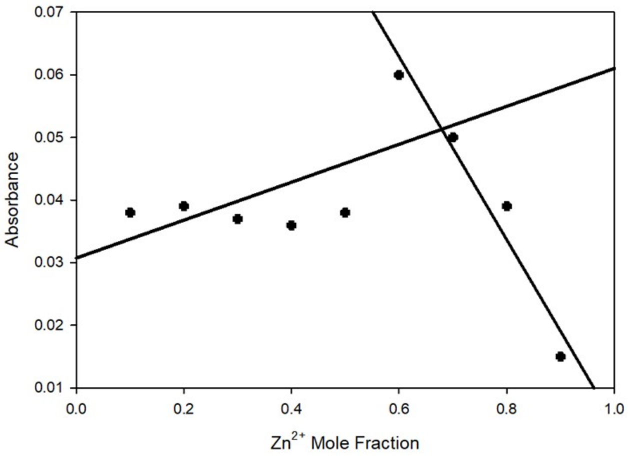

Pyrazole (7), [7] +[Zn^{2+}] = 100 µM, MeCN

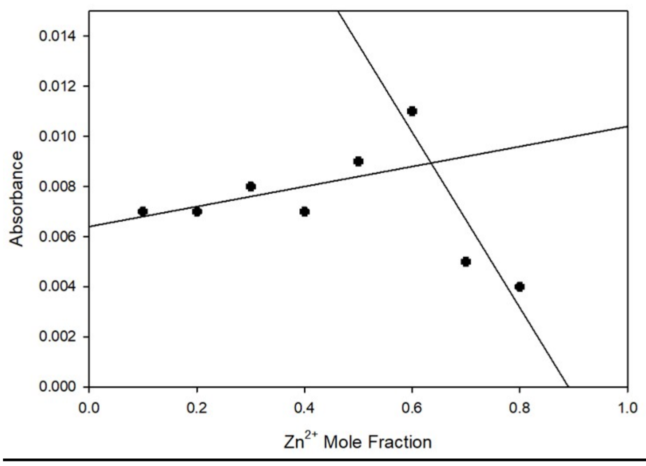

Pyrazole (8), [8] +[Zn^{2+}] = 100 µM, MeCN

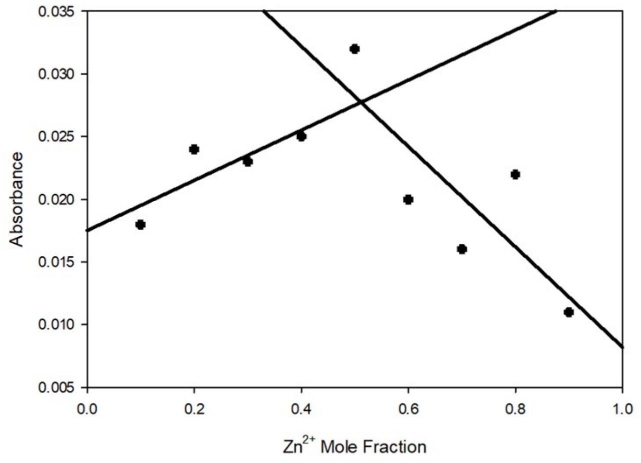

Pyrazole (9), [9] +[Fe^{3+}] = 100 µM, MeCN

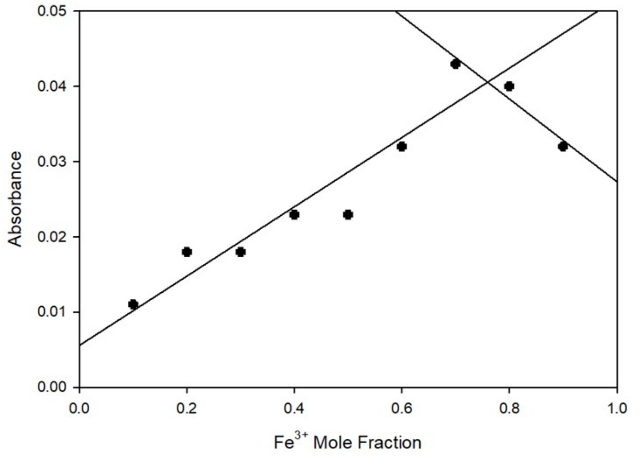

Fluorescence Spectra (S6)

Pyrazoline (4) Metal Screen, λ_{ex}370 nm, 20 µM 4, 100 µM metal, MeCN

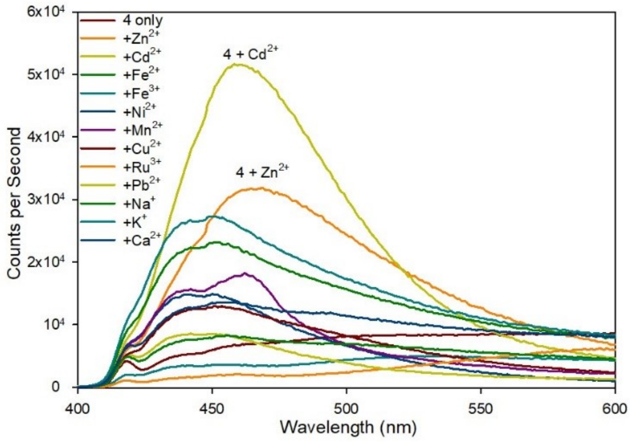

Pyrazoline (5) Metal Screen, λ_{ex}370 nm, 20 µM 5, 100 µM metal, MeCN

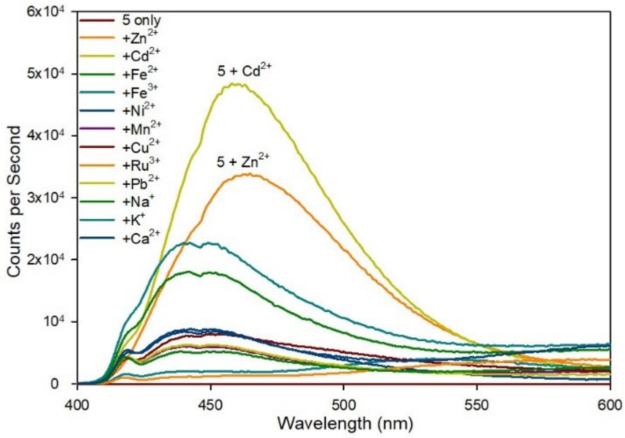

Pyrazoline (6) Metal Screen, λ_{ex}370 nm, 20 µM 6, 100 µM metal, MeCN

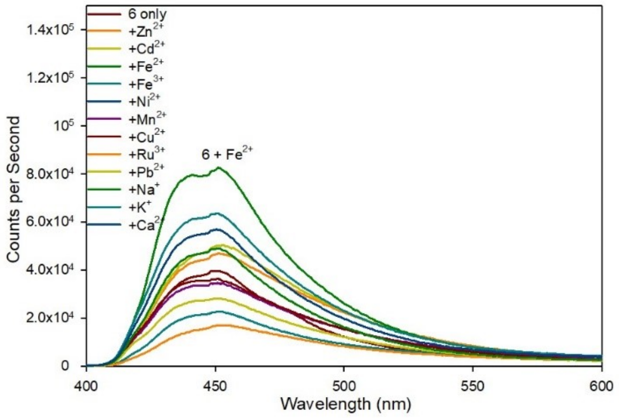

Pyrazole (7) Metal Screen, λ_{ex}290 nm, 20 µM 7, 100 µM metal, MeCN

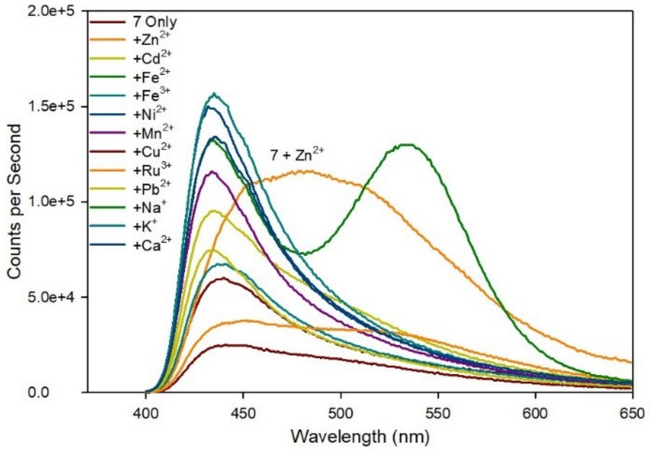

Pyrazole (8) Metal Screen, λ_{ex}290 nm, 20 µM 8, 100 µM metal, MeCN

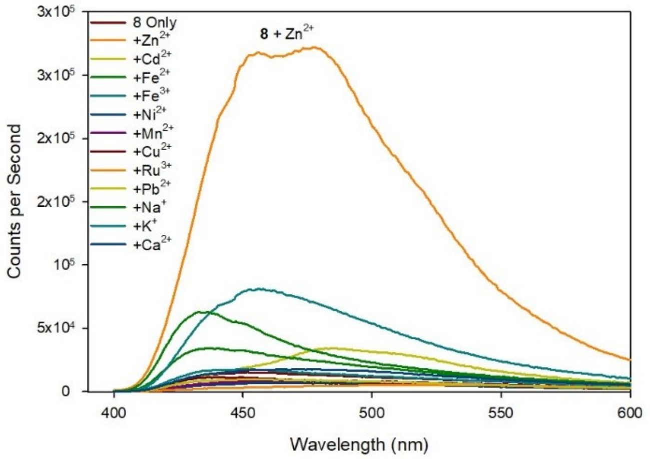

Pyrazole (9) Metal Screen, λ_{ex}290 nm, 20 µM 9, 100 µM metal, MeCN

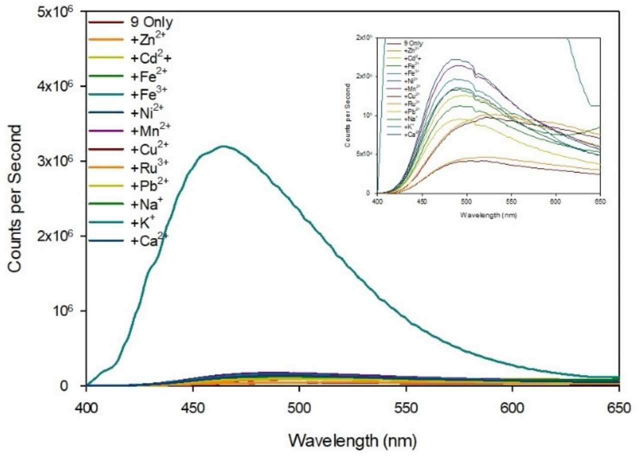

Pyrazoline (4) 20 μM + Zn^{2+} Titration, λ_{ex}370 nm, λ_{em} 480 nm

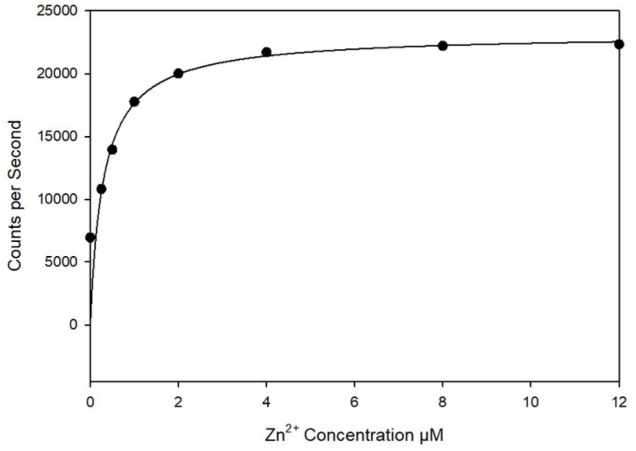

Pyrazoline (5) 20 μM + Zn^{2+} Titration, λ_{ex}370 nm, λ_{em} 480 nm

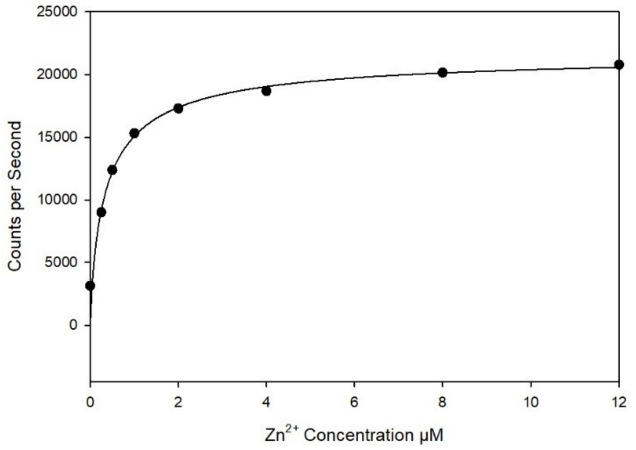

Pyrazoline (6) 20 μM + Zn^{2+} Titration, λ_{ex}370 nm, λ_{em} 480 nm

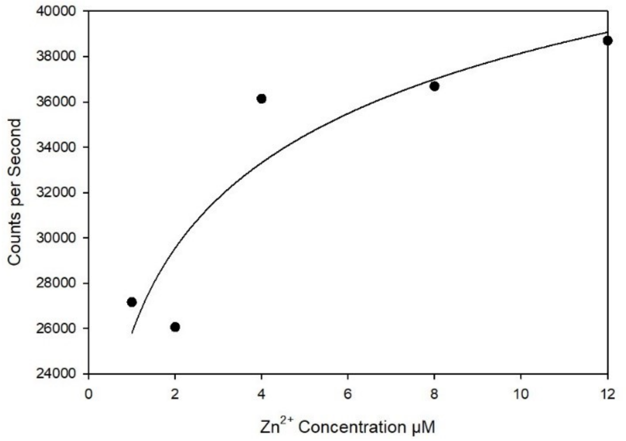

Pyrazole (7) 20 μM + Zn^{2+} Titration, λ_{ex}290 nm, λ_{em} 480 nm

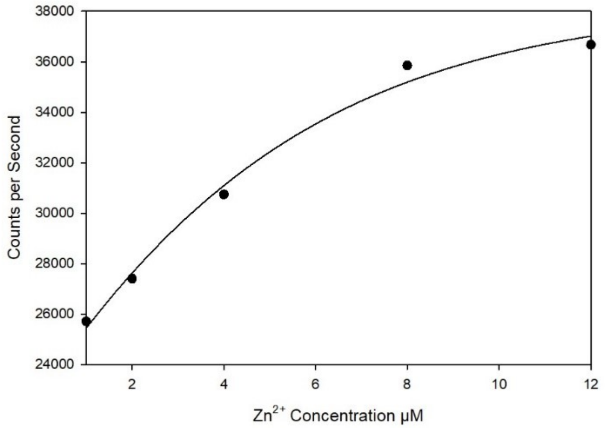

Pyrazole (8) 20 μM + Zn^{2+} Titration, λ_{ex}290 nm, λ_{em} 480 nm

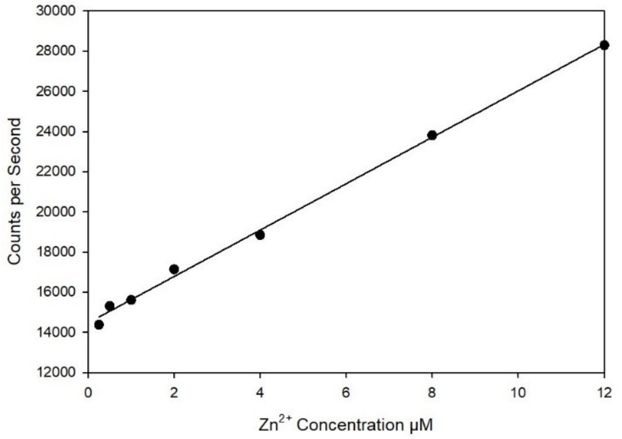

Pyrazole (9) 20 μM + Zn^{2+} Titration, λ_{ex}290 nm, λ_{em} 480 nm

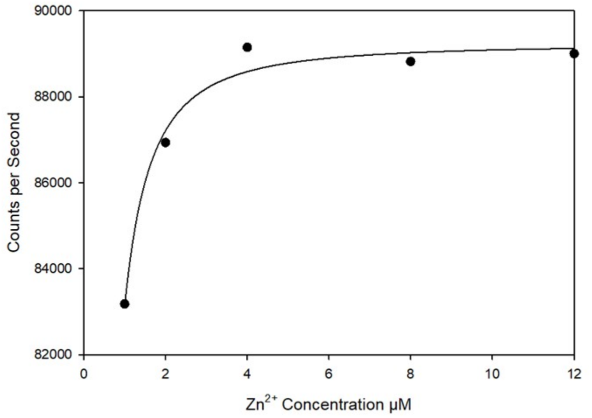

Limit of Detection Calculations (S7)

The method reported by Lee *et al* was used to calculate limit of detection (LoD) for **4-9** in MeCN using the indicated cation with the average from three replicates used. For **6** + Zn^{2+} and **9** +Zn^{2+} an acceptable line of best fit could not be generated therefore detection limit > the highest used cation concentration was given at LoD.

B. P. Joshi, J. Park, W. I. Lee and K.-H. Lee, *Talanta,* 2009, **78**, 903.

Pyrazoline (4) 20 µM, λ_{ex}370 nm, λ_{em} 480 nm

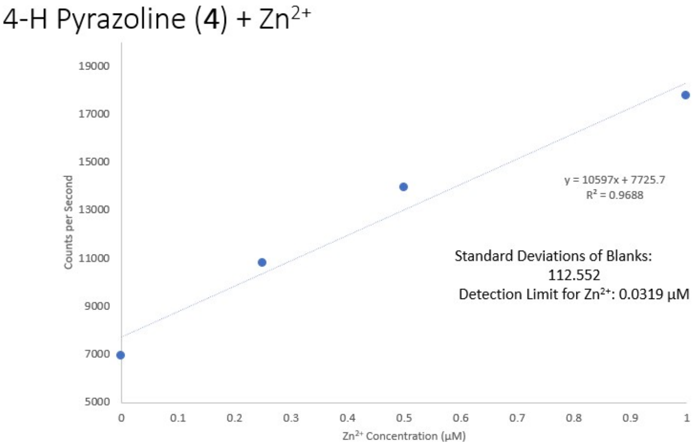

Pyrazoline (5) 20 µM, λ_{ex}370 nm, λ_{em} 480 nm

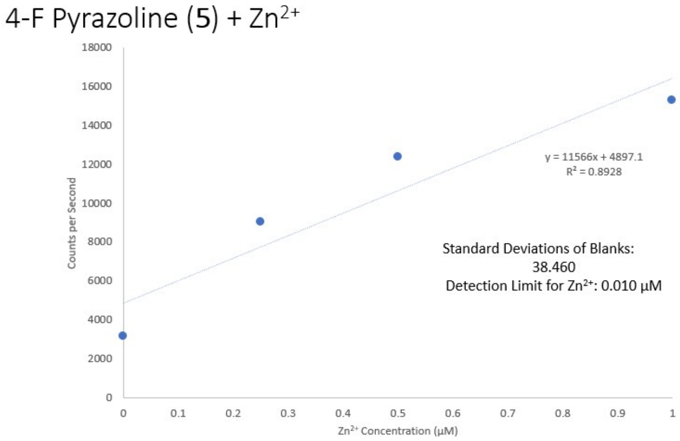

Pyrazoline (6) 20 µM, λ_{ex}370 nm, λ_{em} 480 nm

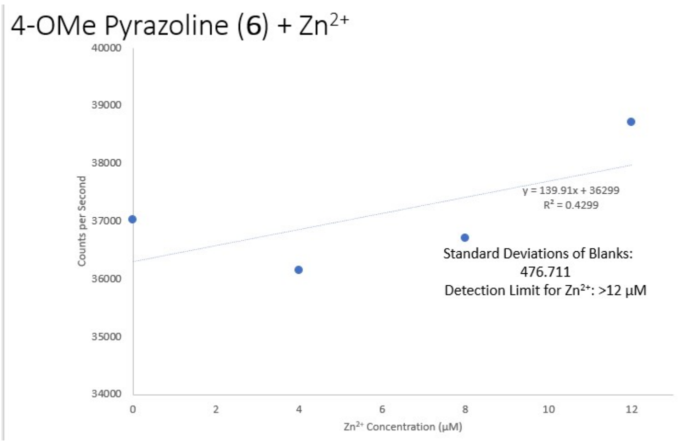

Pyrazole (7) 20 µM, λ_{ex}290 nm, λ_{em} 480 nm

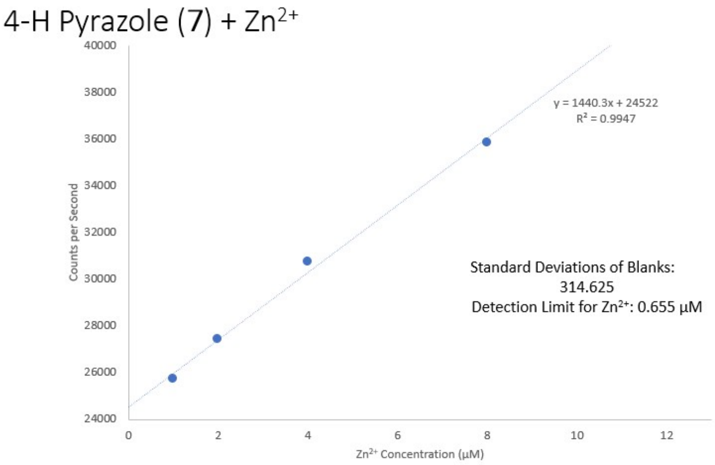

Pyrazole (8) 20 µM, λ_{ex}290 nm, λ_{em} 480 nm

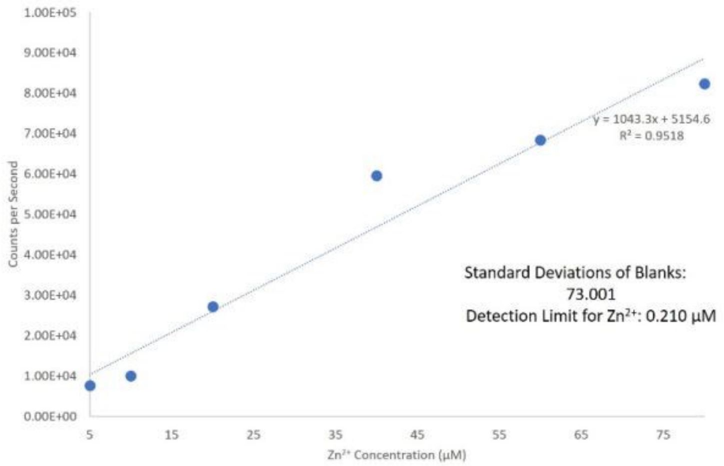

Pyrazole (9) 20 µM for Zn^{2+} λ_{ex}290 nm, λ_{em} 480 nm, for Fe^{3+} λ_{ex}290 nm, λ_{em} 465 nm

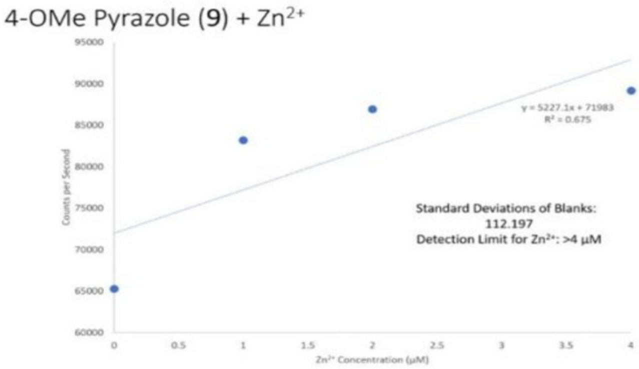

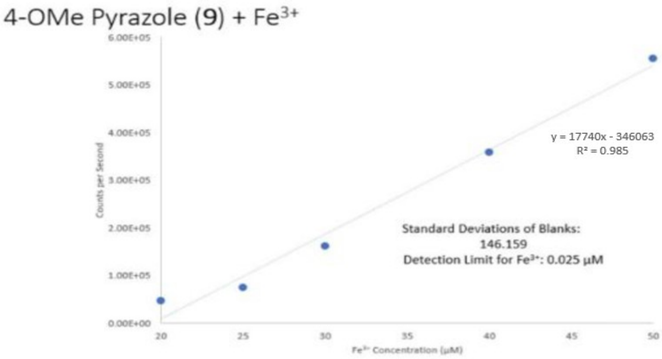

Supplement: RA-014-D4RA00036F-s001 [file RA-014-D4RA00036F-s001.pdf]
